# Characterization of four peptides from milk fermented with kombucha cultures and their metal complexes—in search of new biotherapeutics

**DOI:** 10.3389/fmolb.2024.1366588

**Published:** 2024-04-04

**Authors:** Justyna Kamińska, Aleksandra Hecel, Joanna Słowik, Agnieszka Rombel-Bryzek, Magdalena Rowińska-Żyrek, Danuta Witkowska

**Affiliations:** ^1^ Institute of Health Sciences, University of Opole, Opole, Poland; ^2^ Faculty of Chemistry, University of Wroclaw, Wroclaw, Poland; ^3^ Institute of Medical Sciences, University of Opole, Opole, Poland

**Keywords:** peptides, bioactivity, copper (II) ions, zinc (II) ions, spectroscopic techniques

## Abstract

The most common skin diseases include eczema, psoriasis, acne, and fungal infections. There is often no effective cure for them. Increasing antimicrobial drug resistance prompts us to search for new, safe, and effective therapeutics. Among such interesting candidates are peptides derived from milk fermented with specific lactic acid bacteria or with kombucha cultures, which are a potential treasure trove of bioactive peptides. Four of them are discussed in this article. Their interactions with zinc and copper ions, which are known to improve the well-being of the skin, were characterized by potentiometry, MS, ITC, and spectroscopic methods, and their cytostatic potential was analyzed. The results suggest that they are safe for human cells and can be used alone or in complexes with copper for further testing as potential therapeutics for skin diseases.

## 1 Introduction

Bioactive peptides can be differentiated into the following: (1) carriers, (2) signaling agents, (3) inhibitors of neurotransmitters, and (4) antimicrobial peptides (Lima and Pedriali Moraes, 2018; Akbarian et al., 2022). Carrier peptides are responsible for the transport of trace elements such as copper, zinc, and manganese into the skin and their uptake by the epithelial cells. Some signaling peptides are used as active ingredients in anti-aging preparations (Zhang and Falla, 2009). They are also able to stimulate the fibroblasts of the skin to produce collagen and fibers. Neurotransmitter-inhibitory peptides are able to support muscle activity (Lima and Pedriali Moraes, 2018). Antimicrobial peptides (AMPs) are small (up to about 100 amino acids), usually cationic peptides isolated from natural sources that exhibit antibacterial, antiviral, antifungal, and even anticancer activities; they are a promising source of alternative agents to address the problem of increasing antibiotic resistance. To date, more than 3,600 AMPs are known, which differ in terms of their biological source, activity, structure, and mechanism of action (Wang et al., 2016). It is known that metal ions can enhance the antimicrobial activity of AMPs by changing their charge and/or structure (Łoboda et al., 2018; Bellotti et al., 2019; Dudek et al., 2023). Moreover, the use of some antimicrobial agents is limited due to their high toxicity and non-specific interactions with other drugs (Gomes da Silva Dantas et al., 2018). In this context, coordination complexes of zinc(II) and copper(II) ions and peptides (or peptidomimetics) seem to be important because of their therapeutic potential and low-to-non-existent side effects due to their low toxicity. In addition, copper, which is bound and transported by peptides, has an influence on wound healing and has antioxidant properties. It plays an important role in skin regeneration and angiogenesis (Salvo and Sandoval, 2022). Both zinc and copper play a crucial role in the human body and are essential for maintaining healthy skin (Borkow, 2014). Zinc influences the regenerative properties of the skin, accelerates wound healing, and increases resistance to bacterial infections. Zinc deficiency leads to numerous non-specific general shifts in metabolism and function, including growth deficiencies, increased infections, and impaired wound healing (King et al., 2015; Zołoteńka-Synowiec et al., 2019). Copper(II) and zinc(II) ions are trace elements that are important for the function of many cellular enzymes (e.g., copper and zinc-superoxide dismutase) (Klotz et al., 2003) and can enhance the antimicrobial activity of the peptides (Łoboda et al., 2018; Bellotti et al., 2019).

Bioactive peptides are often not only effective against bacterial and fungal pathogens but also have anti-biofilm, antiviral, anti-cancer, anti-inflammatory, and wound therapeutic effects. The production of these small protein fragments in fermented foods is well-documented and of great interest due to their versatile multifunctional properties (Martinez-Villaluenga et al., 2017; Mohammadian et al., 2017; Peighambardoust et al., 2021).

**FIGURE 1:**
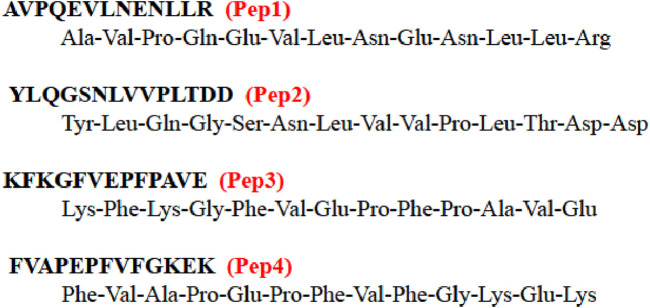
Amino acid sequences of the studied peptides. The short names of these peptides used within this work are represented in red.

In this work, we investigate the coordination abilities of four carefully selected peptides toward zinc(II) and copper(II) and their cytotoxic activity on human dermal fibroblasts and cancer cells with the aim of selecting the peptides and their metal complexes that are good compounds for further testing. The sequences of these peptides were selected from those found after the fermentation of milk by kombucha cultures (Elkhtab et al., 2017). They are shown in Figure 1. Two of these peptides are composed of the same amino acids but in a different order: KFKGFVEPFPAVE (Pep3) and FVAPEPFVFGKEK (Pep4). We were interested in how these sequences influence metal coordination properties and cytotoxicity toward cells.

Kombucha is a beverage derived from the fermentation of sweetened black or green tea and contains polyphenols, amino acids, caffeine, volatile compounds, and minerals added to the biofilm of a symbiotic colony of many non-pathogenic bacteria and yeasts (of the genera *Brettanomyces* and *Saccharomyces*) called SCOBY—symbiotic cultures of bacteria and yeasts (Teoh et al., 2014; Soares et al., 2021). Kombucha is believed to reduce the risk of various cancers, prevent circulatory problems, strengthen the immune system, reduce inflammation, and have a positive effect on the skin, hair, and nails (Elkhtab et al., 2017). The peptides obtained by fermenting milk with SCOBY were suspected to have an inhibitory effect on the angiotensin-converting enzyme, and for some of them, this effect has been confirmed (Peighambardoust et al., 2021). We decided to characterize four synthetic analogs of these peptides: AVPQEVLNENLLR, YLQGSNLVVPLTDD, KFKGFVEPFPAVE, and FVAPEPFVFGKEK (named by us Pep1, Pep2, Pep3, and Pep4, respectively), taking into account their potential to coordinate Cu(II) and Zn(II) ions and test the cytotoxic effect of the Pep1–Pep4 peptides on human dermal fibroblasts (HDFs) and two selected cancer cell lines (the human breast cancer cell line MDA-MB-231 and the human pancreatic cancer cell line PANC-1). The affinity of the interactions of Zn(II) and Cu(II) with the studied peptides (Pep1–Pep4), the changes in enthalpy and entropy, and the stoichiometry of the obtained complexes were determined using isothermal titration microcalorimetry (ITC), potentiometry, mass spectrometry (MS), and spectroscopic techniques (Witkowska and Rowińska-Żyrek, 2019).

## 2 Materials and methods

All the ligand–unprotected peptides (AVPQEVLNENLLR, YLQGSNLVVPLTDD, KFKGFVEPFPAVE, and FVAPEPFVFGKEK) were purchased from KareBay Biochem (United States) (certified purity of 98.00%) and used as received. The purity was checked potentiometrically. Cu(ClO_4_)_2_ was an extra-pure product (Sigma-Aldrich). The carbonate-free stock solution of 0.1 M NaOH was purchased from Merck and then standardized potentiometrically with potassium hydrogen phthalate.

### 2.1 Potentiometric measurements

The stability constants for proton Cu(II) and Zn(II) complexes with four ligands were calculated from titration curves carried out over the pH range of 2–11 at 298 K and ionic strength 0.1 M NaClO_4_. The total volume of the solution used was 3.0 cm^3^. The potentiometric titrations were performed using a Dosimat 800 Metrohm Titrator connected to a Metrohm 905 pH meter and a Mettler Toledo pH InLab Science electrode. The thermostabilized glass cell was equipped with a magnetic stirring system, a microburet delivery tube, and an inlet–outlet tube for argon. The solutions were titrated with 0.1 M carbonate-free NaOH. The electrodes were calibrated daily for a hydrogen ion concentration through titrating HClO_4_ with NaOH using a total volume of 3.0 cm^3^. The ligand concentration was 0.5 mM, and the ratio of metal-to-ligand was 0.9:1. The exact concentrations and purities of the ligand solutions were determined using the Gran method. The standard potential and the slope of the electrode pair were calculated using the GLEE program (Gans and O'Sullivan, 2000). The HYPERQUAD 2008 program was used to calculate the stability constants. The speciation diagrams were calculated using the HYSS program.

### 2.2 Mass spectrometry

High-resolution mass spectra were obtained on a Bruker compact QTOF (Bruker Daltonik, Bremen, Germany) equipped with an electrospray ionization source with an ion funnel. The mass spectrometer was operated in the positive ion mode. The instrumental parameters were as follows: scan range m/z 100–2000, dry gas—nitrogen, temperature 453 K, and ion energy 5 eV. The capillary voltage was optimized to the highest S/N ratio, and it was 4,800 V. The samples were prepared in a 1:1 MeOH:H_2_O mixture at pH 6 with an M:L molar ratio of 0.9:1, where [ligand]_tot_ = 0.1 mM. The samples were infused at a flow rate of 3 μL min^−1^. The instrument was calibrated externally with a Tunemix™ mixture (Bruker Daltonik, Germany) in the quadratic regression mode. Data were processed by application of Compass DataAnalysis 4.2 (Bruker Daltonik, Germany) program. The mass accuracy for the calibration was better than 5 ppm, enabling, together with the true isotopic pattern (using SigmaFit), an unambiguous confirmation of the elemental composition of the obtained complex.

### 2.3 Spectroscopic studies

The absorption spectra were recorded on a Jasco 730 spectrophotometer in the range of 200–800 nm using a quartz cuvette with an optical path of 1 cm. Circular dichroism spectra were recorded on a Jasco J-1500 CD spectrometer in the 200–800 nm range using a quartz cuvette with an optical path of 1 cm in the visible and near-UV range. The concentration of sample solutions used for spectroscopic studies was similar to that employed in the potentiometric experiment. The metal:ligand ratio was 0.9:1. All spectroscopic measurements were recorded in the pH range of 3–11. The pH of the samples was adjusted with the appropriate amounts of HClO_4_ and NaOH solutions. The UV-vis and CD spectroscopy parameters were calculated from the spectra obtained at the pH values corresponding to the maximum concentration of each particular species based on the distribution diagrams. OriginPro 2016 was used to process and visualize the spectra obtained.

### 2.4 Isothermal titration calorimetry

ITC measurements were carried out at 25°C with a MicroCal PEAQ ITC system. All reagents were >99% pure and obtained from Sigma-Aldrich. The peptides were dissolved directly into the 25 mM MES buffer solution at pH 6.1 to mimic the pH of human skin. Metal stock solutions (copper(II) nitrate trihydrate and zinc(II) nitrate hexahydrate) were prepared in deionized water (18 MΩ) at low pH (∼2) and used to prepare 100 mM 2-(N-morpholinic) ethane-sulfonic acid (MES) buffer solutions. After stabilization of the device at 25°C, 40 µL of a metal buffer solution (2 mM for copper and 6 mM for zinc ions) was used to titrate 200 µL of a peptide buffer solution, whose concentration was initially 10 times less than that of the metal ion. Each titration consisted of 19 successive injections, with an interval of 150 s between each aliquot and a stirring speed of 750 rpm, which was repeated few times. The heat of a dilution from a corresponding control titration was subtracted before data fitting. An initial injection of 0.4 µL was discarded from each data set to remove the effect of titrant diffusion across the syringe tip during the equilibration process. A CaCl_2_–EDTA titration was performed at regular intervals for comparison with the results of the initial calibration of the device. The data were processed using MicroCal PEAQ-ITC Analysis Software.

### 2.5 Cytotoxicity experiments

#### 2.5.1 Cell cultures and treatment

The normal human dermal fibroblast (NHDF) (C-12302) line was obtained from PromoCell (Heidelberg, Germany). The human breast cancer cell line MDA-MB-231 (ATCC-HTB-26) and the human pancreatic cancer cell line PANC-1 (ATCC-CRL-1469) were obtained from the American Type Culture Collection (ATCC distributor: LGC Standards, Łomianki, Poland).

NHDF, as well as MDA-MB-231 and PANC-1, were used for the cytotoxicity tests.

Cells were cultured in Dulbecco’s modified Eagle's medium with nutrient mixture F12 (DMEM/F12 1:1) (Corning, United States) and supplemented with 10% fetal bovine serum (FBS) (Capricorn Scientific, Germany). Cells were maintained in a humidifier incubator with a CO_2_ concentration of 5% at 37°C. The cells in the logarithmic growth phase were used for further experiments. Cells were seeded in 96-well culture plates (5 × 10^3^ cells/well) and initially cultured for 24 h before experiments. Subsequently, the original cell culture medium was removed, and the cells were washed twice with PBS. Fresh medium without serum that contained the peptide under investigation at different concentrations (100–500 µM) was added. A medium without peptide was used in the control wells.

#### 2.5.2 Resazurin reduction assay

The resazurin reduction assay to determine cell viability and metabolism was performed according to Ivanov et al. (2014) with modifications. In brief, a stock solution (600 µM in PBS) was aliquoted and stored at −18°C. On the day of analysis, a working solution of 60 µM resazurin in the DMEM/F12 medium was prepared and protected from light. After 24 h of treatment, the medium in the wells was replaced with a working solution of resazurin (100 µL), and the plates were again stored at 37°C. Absorbance was measured at 570 and 600 nm using a microplate reader (BioTek Instruments, United States) 30 min and 4 h after the addition of the dye.

## 3 Results and discussion

### 3.1 Evaluation of the bioactivity of the analyzed peptides using bioinformatic tools

The properties of the peptides that give them specific biological activities are a complex combination of hydrophobicity, net charge, number of amino acids, and other physicochemical properties that can provide useful information for the classification, prediction, and synthesis of new peptides (Fjell et al., 2012). The naturally kombucha-hydrolyzed peptides that were selected are 13–14 amino acid residues long. An online database was used to determine their hydrophobicity, Boman index, and net charge [Antimicrobial Peptide Database (unmc.edu)] (Wang et al., 2016).

**FIGURE 2:**
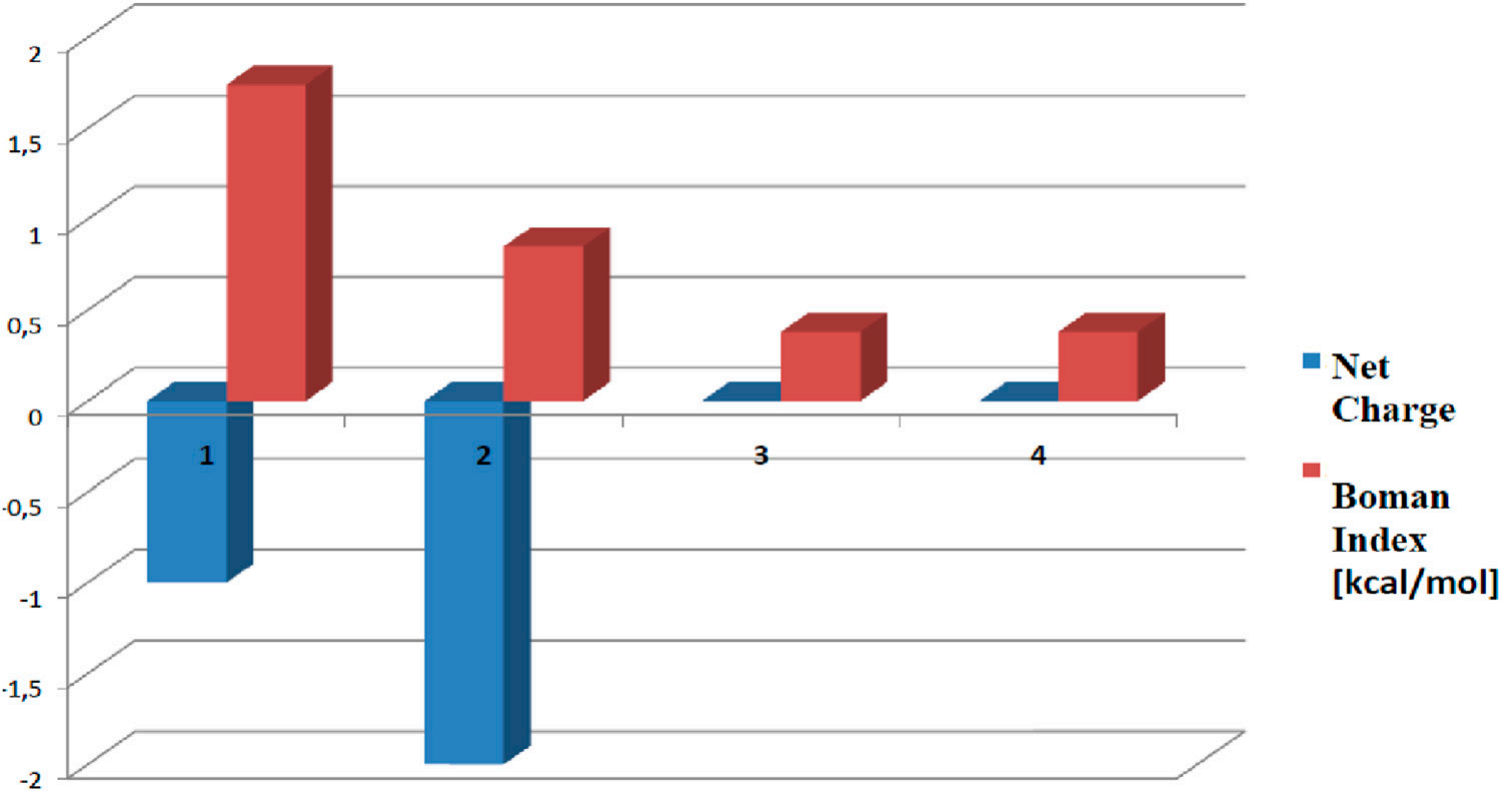
Boman index (in red) and the net charge (in blue) of Pep1–Pep4 calculated with the tools of the Antimicrobial Peptide Database (Wang et al., 2016). Peptides with higher Boman index values are generally considered to be more bioactive as they are more likely to interact favorably with membranes. The net charge of a peptide can also influence its biological properties and interactions with other molecules. The numbers 1, 2, 3, and 4 represent the peptides AVPQEVLNENLLR, YLQGSNLVVPLTDD, KFKGFVEPFPAVE, and FVAPEPFVFGKEK, respectively.

Pep1 and Pep2 are negatively charged (net charge of −1 and −2, respectively), and Pep3 and Pep4 have a net total charge = 0 (Figure 2). The highest Boman index (Boman, 2003) (indicating the total potential of a peptide to bind to other proteins or membranes) is achieved by Pep1 (1.74 kcal/mol). The hydrophobicity of all four peptides ranges from 36% to 46%. The potential allergenicity of all investigated peptides was predicted with AllergenFP (http://ddg-pharmfac.net/AllergenFP/) (Dimitrov et al., 2013). Pep1–3 have been classified as probable non-allergens. Surprisingly, AllergenFP revealed that Pep4 may be a probable allergen. ToxinPred (Gupta et al., 2013) (https://webs.iiitd.edu.in/raghava/toxinpred/index.html) was used to predict the potential toxicity of Pep1–Pep4 peptides. The result of the prediction for all peptides was “non-toxic.” The influence of the amino acid composition of the peptides on the antioxidant activity has been highlighted in the literature. The four peptides studied contain supposedly antioxidant amino acids such as Phe, Lys, Ala, Pro, Leu, and Glu or Asp. These amino acid residues predominate in peptides Pep3 and Pep4, suggesting that they can serve as free radical scavengers (Zou et al., 2016; Ji et al., 2020).

Pep3 and Pep4 also have a theoretically greater potential to serve as AMPs (since Pep1 and Pep2 are negatively charged, which is not common in AMPs). On the other hand, Pep1 is more likely to bind to multiple proteins (it has the highest Boman index) and may exhibit multifunctional behavior (Fjell et al., 2012).

In order to fully understand the interactions of the tested peptides with copper and zinc ions, we analyzed them using a wide range of techniques.

### 3.2 Zn(II) and Cu(II) complexes of Pep1–4

All mass spectrometry (ESI-MS) and spectroscopic results, as well as the distribution diagrams, are shown in Supplementary Information (Supplementary Figures S1–S12).

#### 3.2.1 AVPQEVLNENLLR (Pep1) complexes with Cu(II) and Zn(II) ions

Mass spectrometry (ESI-MS) was used to determine the stoichiometry of Cu(II) and Zn(II) complexes with Pep1–Pep4 peptides, revealing only equimolar complex species. The most abundant peaks with m/z at 758.91 and 513.61 in the Cu(II)–AVPQEVLNENLLR spectrum have been assigned to the sodium adducts [L+Na]^2+^ and [L+2Na]^3+^, respectively. Signals from the copper complex [CuL]^2+^ are present at 777.89 m/z. In the case of the ZnL system, signals from the complexes are much less intense than those observed in the case of the copper complexes. Peaks observed at 778.89 and 547.28 correspond to [ZnL]^2+^ and [ZnL]^3+^, respectively (Supplementary Figures S1, S2). Experimental and simulated spectra for the chosen signals were in perfect agreement for metal complexes of all peptides under study. Other peaks come from differently charged species and from their sodium and potassium adducts.

Based on potentiometric titrations, four protonation constants were established for the Pep1 peptide. The first pK_a_ value 2.51 can be assigned to the deprotonation of the carboxyl group at the C-terminus. The next two (pK_a_ 4.84 and 3.88) are related to the deprotonation of two glutamic acid residues, and the most basic one (7.94) corresponds to the deprotonation of the amine group of the N-terminus (Table 1) (Pettit et al., 1991).

**TABLE 1:** Potentiometric and spectroscopic data for proton and Cu(II) complexes with Pep1.

Species	Logβ	pKa	UV-Vis		CD		Proposed donors
			λ [nm]	ε [cm^−1^M^−1^]	λ [nm]	Θ [M^−1^cm^−1^]	
HL	7.94 (2)	7.94 (N-terminal NH_3_^+^)					
H_2_L	12.78 (3)	4.84 (E)					
H_3_L	16.66 (3)	3.88 (E)					
H_4_L	19.17 (5)	2.51 (C-terminal COOH)					
CuH_2_L	17.65 (7)						
CuHL	13.75 (5)	3.90					E
CuL	8.73 (6)	5.02			274	−0.08	NH_2_
					314	0.10	
CuH_-1_L	2.81 (5)	5.92	662	59	268	−0.64	NH_2_,1N^−^
					314	0.81	
					696	−0.43	
CuH_-2_L	−3.45 (6)	6.26	657	85	268	−0.97	NH_2_,2N^−^
					314	1.30	
					694	−0.70	
CuH_-3_L	−12.52 (6)	9.07	620	83	274	−0.27	NH_2_,3N^−^
					310	0.86	
					518	−0.45	
					712	−0.46	
CuH_-4_L	−22.28 (6)	9.76	590	74	258	2.04	4N^−^
					316	0.40	
					510	−0.74	
					622	0.24	
					732	−0.19	

Titrations were carried out over the pH range 2–11 at T = 298 K in an aqueous solution with 4 mM HClO_4_ and 0.1 M NaClO_4_. Standard deviations are shown in brackets.

Cu(II)–Pep1 shows seven complex forms. The first acidic form (CuH_2_L) appears at a pH below 3. The next (CuHL) achieves its maximum at about pH 4.5 and comes from deprotonation of an glutamic acid residue, which may participate in Cu(II) binding (pK_a_ = 3.90 compared to a protonation constant of 4.84). In the CuL form (maximum at pH 5.5), the N-terminal amine is the anchoring site in the Cu(II)–AVP complex. In addition to the significant lowering of pK_a_ (5.02) values of the CuL complex compared to the free ligand (pK_a_ 7.94), the coordination of the free amine group provides a band at 274 nm on CD spectra, which corresponds to (charge transfer) CT between the Cu(II) → NH_2_ group (Supplementary Figure S1). The next three species (CuH_-1_L, CuH_-2_L, and CuH_-3_L) results from the coordination of three amide groups. The blue shift of the maximum from 662 to 620 nm on UV-Vis (Supplementary Figure S1) spectra provides a coordination of the increasing number of nitrogen atoms to Cu(II). The appearance of two bands on CD spectra (518 and 712 nm) confirms that a square–planar complex forms at a pH above 9 (CuH_-3_L form) (Miller et al., 2021). The binding of four nitrogen atoms is also indicated by the absorption spectrum, where the maximum at pH 9.90–11 moves to 590 nm. The last CuH_-4_L, with the maximum at pH 11, results from the deprotonation of the fourth amide group, which replaces the N-terminal amine group from the metal binding, forming a 4N^−^ or 3Ni^−^ and one water molecule complex (Table 1, Supplementary Figure S3A).

Zn(II)–Pep1 complexes are found in five different protonation states. Zn(II) starts to interact with pep1 at pH below 3, resulting in a ZnH_2_L form, in which carboxylate side chains could be the anchoring sites for Zn(II). The next species, ZnHL (maximum concentration at pH 4.5) with pK_a_ = 4.10, corresponds to the deprotonation of an glutamic acid residue, which may contribute to Zn(II) binding (the pK_a_ value of this residue in the free peptide is 4.85). The following complex (ZnL), with pK_a_ = 4.70, dominates over the pH range 5–8, with a maximum at 6.5. This form includes a coordination of the N-terminal amine (the protonation constant in the free peptide is 7.94; a significant difference between the protonation and stability constants indicates the coordination of the group). The next two species (ZnH_−2_L and ZnH_−3_L), arising from deprotonation of water ligands of the Zn(II)–peptide complex (pK_a_ 7.88), fill the preferred tetrahedral zinc coordination sphere 19 (Valensin et al., 2009), (Supplementary Table S1; Supplementary Figure S3B).

#### 3.2.2 YLQGSNLVVPLTDD (Pep2) complexes with Cu(II) and Zn(II) ions

On the basis of a series of potentiometric titrations, five protonation constants were established. Three of them are assigned to the carboxyl groups of two Asp residues (p*K*_*a*_ 3.92 and 4.95) and the carboxyl group of C-terminus (pKa 2.54). The next pKa value (7.21) corresponds to the deprotonation of the amine group at the N-terminus. The most basic one (pKa 9.71) was assigned to the tyrosine residue (Table 2).

**TABLE 2:** Potentiometric and spectroscopic data for proton and Cu(II) complexes with Pep2.

Species	Logβ	pKa	UV-Vis		CD		Proposed donors
			λ [nm]	ε [cm^−1^M^−1^]	λ [nm]	Θ [M^−1^cm^−1^]	
HL	9.71 (1)	9.71(Y)					
H_2_L	16.92 (2)	7.21(N-terminal NH_3_^+^)					
H_3_L	21.87 (3)	4.95(D)					
H_4_L	25.79 (3)	3.92(D)					
H_5_L	28.33 (5)	2.54(C-terminal COOH)					
CuH_2_L	20.27 (2)						
CuHL	14.71 (2)	5.56	648	107	288	−0.14	NH_2_
					320	0.21	
CuL	8.54 (2)	6.17	624	144	288	−0.31	NH_2_,1N^−^
					320	0.36	
CuH_-1_L	1.39 (2)	7.15	507	208	268	−0.69	NH_2_,2N^−^
					310	0.54	
					518	−0.73	
CuH_-2_L	−5.81 (2)	7.20	499	238	246	0.79	NH_2_,3N^−^
					270	−0.90	
					304	0.70	
					512	−1.23	
CuH_-3_L	−15.17 (3)	9.36	497	263	264	−1.82	NH_2_,3N^−^/4N^−^
					298	1.31	
					512	−1.33	

Titrations were carried out over the pH range 2–11 at T = 298 K in an aqueous solution with 4 mM HClO_4_ and 0.1 M NaClO_4_. Standard deviations are shown in brackets.

From the species distribution diagram, it appears that the copper ion interacts with the Pep2 peptide from pH 3, most likely via the carboxyl group (the CuH_2_L complex), as suggested by the stoichiometry of this species. The oxygen atoms are not favorable donors for Cu(II) ions, but a weak interaction, especially at acidic pH, may occur. The next (CuHL) achieves its maximum at approximately pH 6 and results from the deprotonation and binding of the N-terminal amine group to Cu(II) ions (significant lowering of pK_a_ values for this complex (pK_a_ 5.56) compared to the free ligand (7.21)). The UV-Vis absorption band at 648 nm may also indicate a 1N binding mode. The involvement of first amide nitrogen in Cu(II) binding is providing for CuL species (pK_a_ 6.17). The maximum concentration of this form is reached at pH 6.8. At this pH, the absorption UV-Vis band at 624 nm confirms the 2N coordination mode, and on CD spectra, the involvement of amide nitrogen is provided by a characteristic band at 520 nm at pH above 7, which is not observed at a lower pH (Supplementary Figure S6A). The second amide group (CuH_−1_L; pK_a_ = 7.15) binds Cu(II) at a pH around 7.5. The absorption spectrum indicates the binding of a second amide (third nitrogen) at pH 8 by a shift of the maximum to 520 nm. The third amide coordinates Cu(II) at around pH 8.5 (CuH_−2_L). The binding of four nitrogen atoms is also indicated by the absorption spectrum, where the maximum at pH 8–11 moves to 497 nm. In the CD spectrum, the intensity of the characteristic peaks at 512 nm stabilizes over the pH range 8.7–11, indicating a rising concentration of the square–planar complex (Supplementary Figure S6A; Table 2). The last CuH_-3_L form results from the coordination of the fourth nitrogen amide, which replaces the amino group from the metal-binding sphere or deprotonation of non-binding tyrosine residues (pK_a_ 9.36).

The first Zn(II)–Pep2 complex form, ZnH_3_L, has a maximum at approximately pH 3.3 and most likely includes at least one carboxyl group in the binding sphere. The loss of one proton (a pK_a_ value of 4.18) leads to the ZnH_2_L form. The pK_a_ value of this aspartic acid is slightly decreased with respect to that of the free ligand (pK_a_ = 4.95), which may suggest that the second carboxyl group is also involved in the coordination. The loss of the next proton leads to the ZnHL form, and it is related to the deprotonation of the N-terminal amine group bound to the central Zn(II) ion (the pK_a_ value 5.07 is significantly decreased with respect to that of the free ligand pKa 7.21). The next two calculated forms (ZnL and ZnH_-2_L) come from water molecules bound to Zn(II). For ZnL (max at pH 7.5), one nitrogen and one water molecule constitute the main binding site, and for ZnH_-2_L (max at 8.7), four coordinated donors are present (NH_2_,3H_2_O). The last observed form, ZnH_-3_L, corresponds to the deprotonation of the tyrosine side chain group and has no impact on the complex coordination mode (Supplementary Table S2; Supplementary Figure S6B).

#### 3.2.3 KFKGFVEPFPAVE (Pep3) complexes with Cu(II) and Zn(II) ions

The KFKGFVEPFPAVE peptide acts like a typical LH_6_ acid. The first three pK_a_ values (2.83, 4.01, and 4.81) come from the deprotonation of three carboxyl groups of the two Glu residues and the C-terminus. The next constant (7.17) corresponds to the deprotonation of the amine group of the N-terminus. The highest two (11.00 and 9.94) are associated with the deprotonation of two lysine residues. Cu(II)–Pep3 has five complex forms, with the first (CuH_2_L) reaching its maximum at around pH 5.2. The corresponding absorption maximum at 675 nm and CD CT band at 271 nm indicate the coordination of one nitrogen atom, which implicates the N-terminal amine as an anchoring site (Supplementary Figure S7). The next two species (CuHL and CuH_-1_L) are due to the coordination of one and three amides, respectively. The NH_2_,1N^−^ and NH_2_,3N^−^ binding modes for these forms (CuHL and CuH_-1_L, respectively) are in good agreement with the UV-Vis spectra indicating on 2N (638 nm) and 4N (499 nm) complexes. The significant intensive negative absorption band at 500 nm in pH approximately 7.5–8 on CD spectra indicates that the square–planar complex is starting to form at this pH (CuH_-1_L). Any significant changes in the UV-Vis and CD spectra above pH 8 indicates the same coordination mode for two last forms (CuH_-2_L, CuH_-3_L), which result from the deprotonation of two lysine residues, which do not participate in the binding (Table 3; Supplementary Figures S7, S9A).

**TABLE 3:** Potentiometric and spectroscopic data for proton and Cu(II) complexes with Pep3.

Species	Logβ	pKa	UV-Vis		CD		Proposed donors
			λ [nm]	ε [cm^−1^M^−1^]	λ [nm]	Θ [M^−1^cm^−1^]	
HL	11.00 (1)	11.00(K)					
H_2_L	20.94 (1)	9.94(K)					
H_3_L	28.11 (2)	7.17(N-terminal NH_3_^+^)					
H_4_L	32.92 (2)	4.81 (E)					
H_5_L	36.93 (2)	4.01 (E)					
H_6_L	39.76 (2)	2.83 (C-terminal COOH)					
CuH_2_L	25.52 (9)		675	69	271	−0.79	NH_2_
					330	0.12	
CuHL	20.53 (1)	4.99	638	105	273	−0.18	NH_2_,1N^−^
					321	0.04	
					618	−0.03	
CuL	-						
CuH_-1_L	7.05 (2)		499	167	270	−2.17	NH_2_,3N^−^
					303	0.6	
					513	−1.10	
CuH_-2_L	−2.41 (4)	9.46	497	175	268	−2.41	NH_2_,3N^−^/4N^−^
					303	0.80	
					518	−1.26	
CuH_-3_L	−13.16 (4)	10.75	497	173	268	−2.41	NH_2_,3N^−^/4N^−^
					303	0.80	
					518	−1.26	

Titrations were carried out over the pH range 2–11 at T = 298 K in an aqueous solution with 4 mM HClO_4_ and 0.1 M NaClO_4_. Standard deviations are shown in brackets.

The Zn(II)–Pep3 complex exists in four different protonated forms. Zn(II) starts to bind to this peptide at a pH of approximately 3, resulting in the ZnHL species (maximum at pH 4.2). The next complex (ZnH_2_L), with pK_a_ = 5.09, dominates over the pH range 5–7 and corresponds to the coordination by the N-terminal amine. From pH 7.5, the coordination of water molecules to Zn(II) starts, resulting in the formation of ZnHL and ZnH_-2_L complex forms (Supplementary Table S3; Supplementary Figure S9B) (Valensin et al., 2009).

#### 3.2.4 FVAPEPFVFGKEK (Pep4) complexes with Cu(II) and Zn(II) ions

In the FVAPEPFVFGKEK peptide, potentiometric measurements were able to detect five protonation constants. The first two pK_a_ values (3.98 and 4.86) come from the deprotonation of the carboxylic side chain of Glu residues. The next one is associated with the N-terminal amino group (pK_a_ 7.27), and the last two calculated pK_a_ values (10.61 and 9.61) correspond to deprotonation of Lys residues. In the studied pH range, the deprotonation of the carboxyl group of the C-terminus could not be detected. The presence in close proximity of two positively charged groups of lysine residues lowers the pK_a_ value of C-terminus, bringing its pK_a_ value close to 2 or even less. Cu(II)–Pep4 exists in eight protonation forms. The first acidic form (CuH_4_L) achieves its maximum at a pH of approximately 3.2, where two carboxyl groups (C-terminus and side chain of Glu) are deprotonated already. For this form, we cannot clearly exclude their participation in Cu(II) binding. The next stability constant CuH_3_L arises from the deprotonation of the second glutamic acid residue, which may constitute the anchoring oxygen donor for Cu(II) ions, as suggested by a slight difference between the stability and protonation constants (4.03 and 4.86 for the complex and free peptide, respectively). The CuH_2_L form results from the deprotonation and Cu(II) binding to one nitrogen atom from the N-terminal amine group. The pK_a_ values for this complex is significantly lowered (pK_a_ 4.84) compared to the free peptide (pKa 7.27). On CD spectra, the CT band at 267 nm indicates the involvement of the amine group in Cu(II) binding. The corresponding absorption maximum at 677 nm indicates the coordination of one nitrogen atom as well (Supplementary Figure S10). The next three species (CuHL, CuL, and CuH_-1_L) are due to the coordination of three amides (pK_a_ = 5.61, 5.78, and 7.83, respectively) (Hecel et al., 2017). For CuHL species, characteristic CD bands in the d–d range appear (527 and 701 nm), which provides the amide nitrogen in Cu(II) binding. The involvement of following 1, 2, and 3 amide nitrogen atoms for CuHL, CuL, and CuH_-1_L forms, respectively, arises from the blue shift from 661→ 579 nm on the UV-Vis spectra. The formation of the square–planar complex for the CuH_-1_L form (maximum concentration at 8.5 pH) confirms the characteristic shift on the CD spectra noticeable from pH 8 at approximately 600 nm. The CuH_-2_L species may result from the coordination of the fourth nitrogen amide (pK_a_ 9.30), which replaces the amino group from the metal-binding sphere, or deprotonation of non-binding lysine residues (Table 4; Supplementary Figure S12A). The last calculated CuH_-3_L form is associated with deprotonation of lysine residues, which do not participate in metal binding (pK_a_ 10.07).

**TABLE 4:** Potentiometric and spectroscopic data for proton and Cu(II) complexes with Pep4.

Species	Logβ	pKa	UV-Vis		CD		Proposed donors
			λ [nm]	ε [cm^−1^M^−1^]	λ [nm]	Θ [M^−1^cm^−1^]	
HL	10.61 (1)	10.61 (K)					
H_2_L	20.22 (1)	9.61(K)					
H_3_L	27.49 (3)	7.27 (N-terminal NH_3_^+^)					
H_4_L	32.35 (4)	4.86 (E)					
H_5_L	36.33 (4)	3.98 (E)					
CuH_4_L	36.77 (4)						
CuH_3_L	32.74 (3)	4.03					
CuH_2_L	27.90 (4)	4.84	677	41	267	−0.45	NH_2_
					315	0.35	
					701	−0.14	
CuHL	22.29 (4)	5.61	661	62	266	−1.01	NH_2_,N^−^
					315	0.84	
					527	−0.11	
					701	−0.29	
CuL	16.51 (4)	5.78	642	101	269	−1.42	NH_2_,2N^−^
					313	1.21	
					553	−0.25	
					683	−0.46	
CuH_-1_L	8.68 (5)	7.83	579	132	248	0.87	NH_2_,3N^−^
					277	−0.96	
					314	1.36	
					601	−0.81	
CuH_-2_L	−0.62 (5)	9.30	567	152	248	1.32	NH_2_,3N^−^/4N^−^
					278	−0.88	
					317	1.30	
					593	−0.99	
CuH_-3_L	−10.69	10.07	567	156	250	0.75	NH_2_,3N^−^/4N^−^
					281	−0.98	
					319	1.29	
					588	−0.87	

Titrations were carried out over the pH range 2–11 at T = 298 K in an aqueous solution with 4 mM HClO_4_ and 0.1 M NaClO_4_. Standard deviations are shown in brackets.

For the Zn(II)–Pep4 complex, six forms are observed. The first could be the carboxylate-bound ZnH_3_L, detected at a pH of approximately 3.5. For this species, all acidic carboxyl groups are deprotonated already; thus, it is very likely that at least one of them may constitute the anchoring site for Zn(II). The ZnH_2_L complex, with the maximum concentration at pH 4.8, involves the N-terminal amine in binding (pK_a_ = 4.27 for the Zn(II) complex and 7.27 for the free peptide). The next two species, ZnHL and ZnL, (pK_a_ = 5.03, 7.91) arise from deprotonation of water ligands that are coordinated to Zn(II). ZnH_-1_L was overlapped by the dominant ZnH_-2_L species; thus, calculating the logβ value of this species was not possible. The last ZnH_-2_L form may result from the coordination of fourth water molecules, forming a tetragonal pyramid or trigonal bipyramid geometry (Supplementary Table S4; Supplementary Figure S12B) (Soares et al., 2021).

### 3.3 Thermodynamic stability confirmed by ITC results

In order to better understand the thermodynamics of the investigated systems at certain pH values, we analyzed the complexes using isothermal microcalorimetry. Since the ITC technique provides results that are conditional, all studies for Cu(II) ions with ITC were performed under the same experimental conditions (MES buffer, pH = 6.1, and temperature 25°C) so that they could be compared (Wyrzykowski et al., 2014).

**FIGURE 3:**
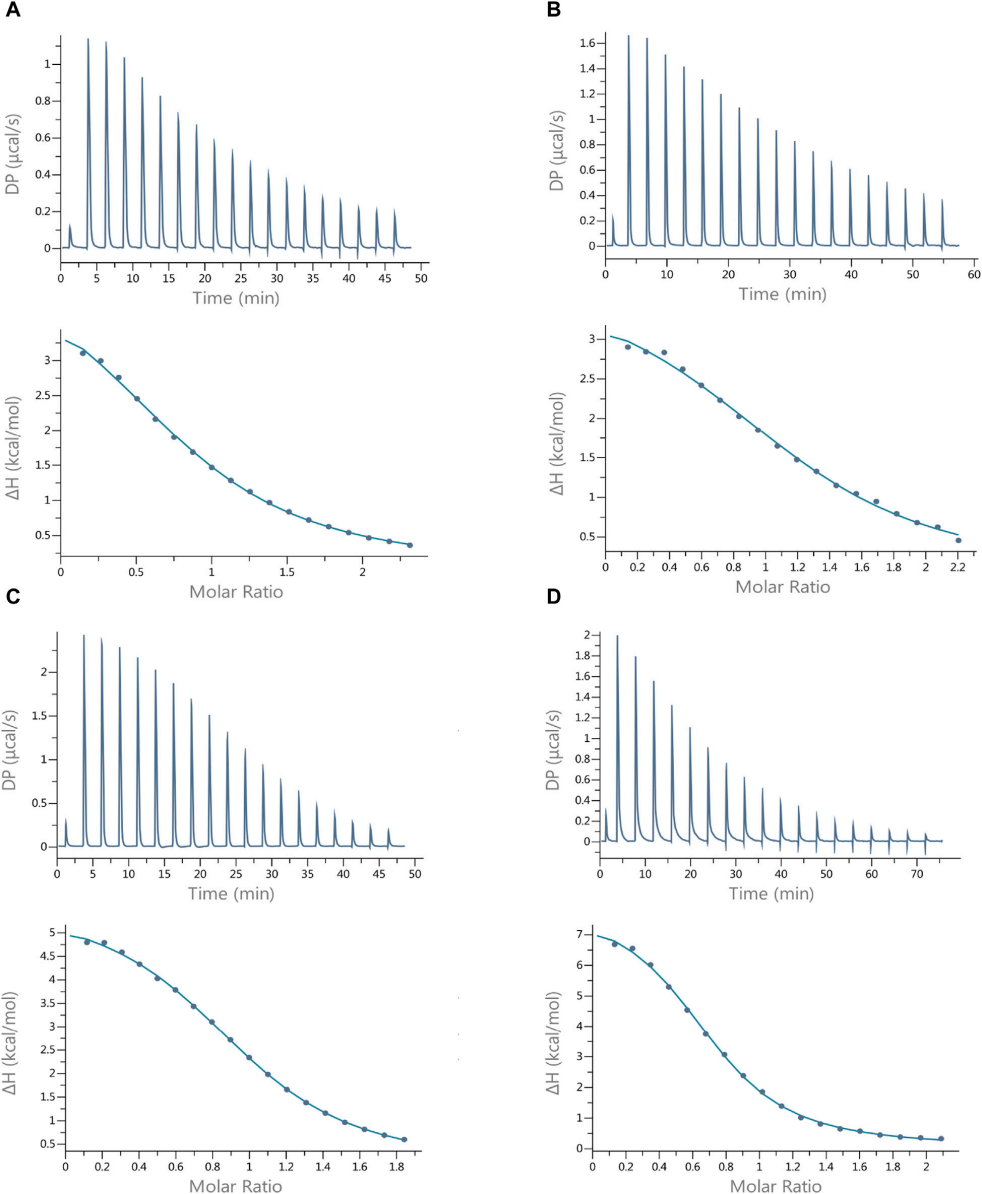
Best-fit ITC data for the 25 °C titration of 3 mM Cu(II) into **(A)** Pep1, **(B)** Pep2, **(C)** Pep3, and **(D)** Pep4 peptides in MES buffer at pH 6.1. The peptide concentration was within the range of 242–304 µM.

The results of the interactions of Cu(II) ions with the peptides studied show that all these reactions are endothermic (∆H positive). The dissociation constants (K_d_) show that all peptides form Cu(II) complexes of moderate thermodynamic stability (Table 5), and the most stable complex with copper ions is formed by Pep4 (K_d_ = 35.7 µM); however, the difference in stability of all the complexes is small. These complexation reactions are entropy-driven for all peptides, and the stoichiometry of the Cu(II) peptide was determined by ITC as 1 (Table 5; Figure 3) (Grossoehme et al., 2010). The upper panels of Figures 3A–D show the heat flow over time during the titration, and the lower panels show the integrated, concentration-normalized enthalpy for each aliquot as a function of the molar ratio of the titrant (metal) to the titrand (peptide) in the reaction cell. The solid line in the bottom panel (Figure 3) shows the best fit to a single-site binding model, what is also confirmed by MS.

**TABLE 5:** Experimental (conditional) thermodynamic values for Cu (II) binding to Pep1, Pep2, Pep3, and Pep4 peptides from ITC measurements in MES buffer at 25°C.

Ligand	$K_{D}\;\left[M\right]$	ΔH [kcal/mol]	– TΔS [kcal/mol]	N [sites]
Pep1	${82,05\;\left(\pm \;7,55\right)\cdot 10\;}^{-6}$	4.59 ± 0.32	−10.2	${0,89\pm 1,9\;\cdot 10}^{-2}$
Pep2	${92,65\;\left(\pm 24,5\right)\;\cdot 10}^{-6}$	3.76 ± 0.43	−9.28	${1,12\;\pm \;5,35\;\cdot 10}^{-2}$
Pep3	${42,4\;\left(\pm 2,55\right)∙10}^{-6}$	5.89 ± 0.11	−11.85	${0,94\;\pm \;0,94\;\cdot 10}^{-2}$
Pep4	${35,7\;\left(\pm 5,14\right)∙10}^{-6}$	7.01 ± 0.28	−13.07	${0,81\;\pm \;0,87\;\cdot 10}^{-2}$

Titration of the same peptides with Zn(II) ions under the same experimental conditions (MES buffer, pH = 6.1, and temperature 25°C) resulted in the formation of much less stable complexes, although the zinc ion concentration was increased from c = 3 mM to c = 6 mM. For this reason, it was not possible to calculate the thermodynamic parameters (K_d_, ∆S, and ∆H) accurately (Turnbull and Daranas, 2003). ITC traces and binding isotherms of titration of the Pep1–Pep4 solutions with Zn(II) ions are shown in Supplementary Figure S13.

Naturally occurring Zn(II)-binding sequences mainly consist of different amounts of His and Cys residues (Rich et al., 2012). Sometimes, copper(II) and zinc(II) ions may compete for binding to the same amino acid residues (identical binding donor atoms) in peptides (Migliorini et al., 2010). There also appears to be a competition between copper(II) and zinc(II) in the human body (Gunturu et al., 2021). In the case of the Pep1–Pep4 peptides, our results show that Cu(II) forms thermodynamically much more stable complexes than Zn(II).

### 3.4 Resazurin reduction–cell metabolism assay

**FIGURE 4:**
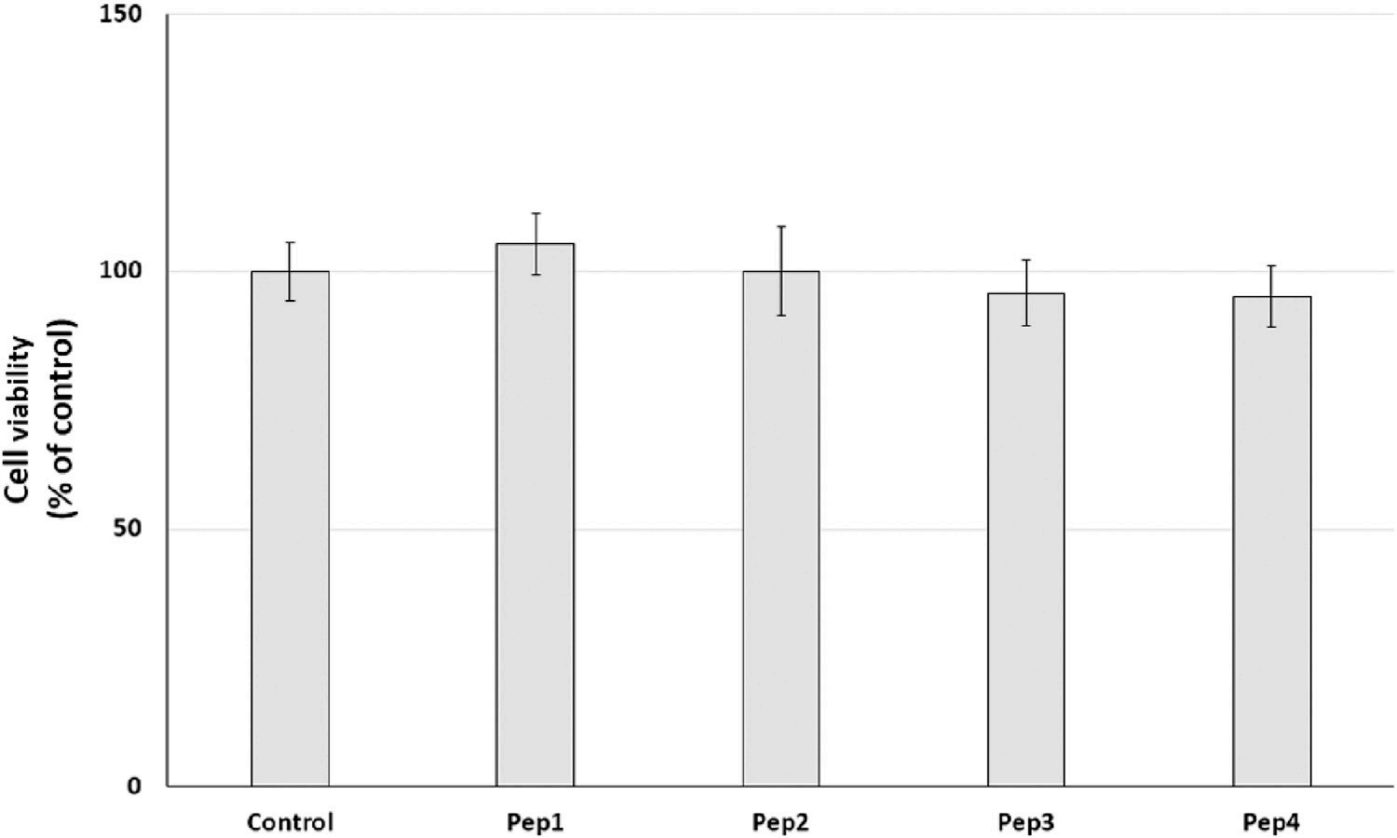
Effect of peptides (200 µM) on cell viability (%) expressed as resazurin reduction in normal human dermal fibroblasts after a 24-h exposure. The bar graph represents the mean and standard deviation of two independent experiments with six replicates each.

Initially, the cytotoxic effect of Pep1–Pep4 peptides on normal human dermal fibroblasts (HDFs) was investigated. NHDFs are mainly found in the dermis and play an important role in the production of the extracellular matrix (ECM) and wound healing (Mesdom et al., 2020). The cells were treated with Pep1–Pep4 at a concentration of 200 µM for 24 h, and their cytotoxicity was determined using a resazurin reduction assay. Resazurin reduction was similar for all peptides tested, with no significant decrease (Figure 4), suggesting that Pep1–Pep4 have no cytotoxic effect on normal human fibroblasts.

**FIGURE 5:**
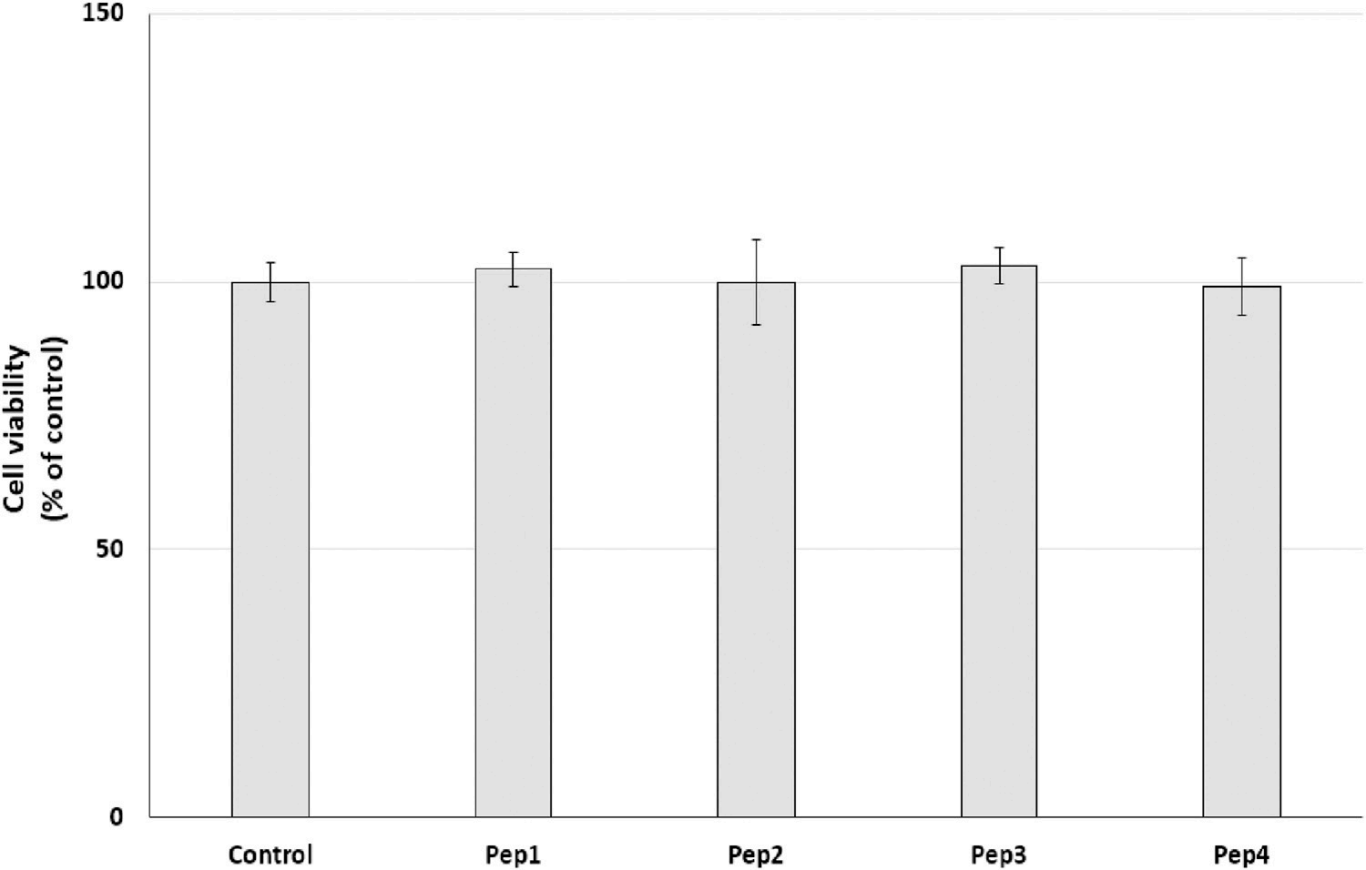
Effect of peptides (200 µM) on cell viability (%) expressed as resazurin reduction in the MDA-MB-231 cell line after a 24-h exposure. Bar graph representing the mean and standard deviation values of two independent experiments, each consisting of six replicates.

**FIGURE 6:**
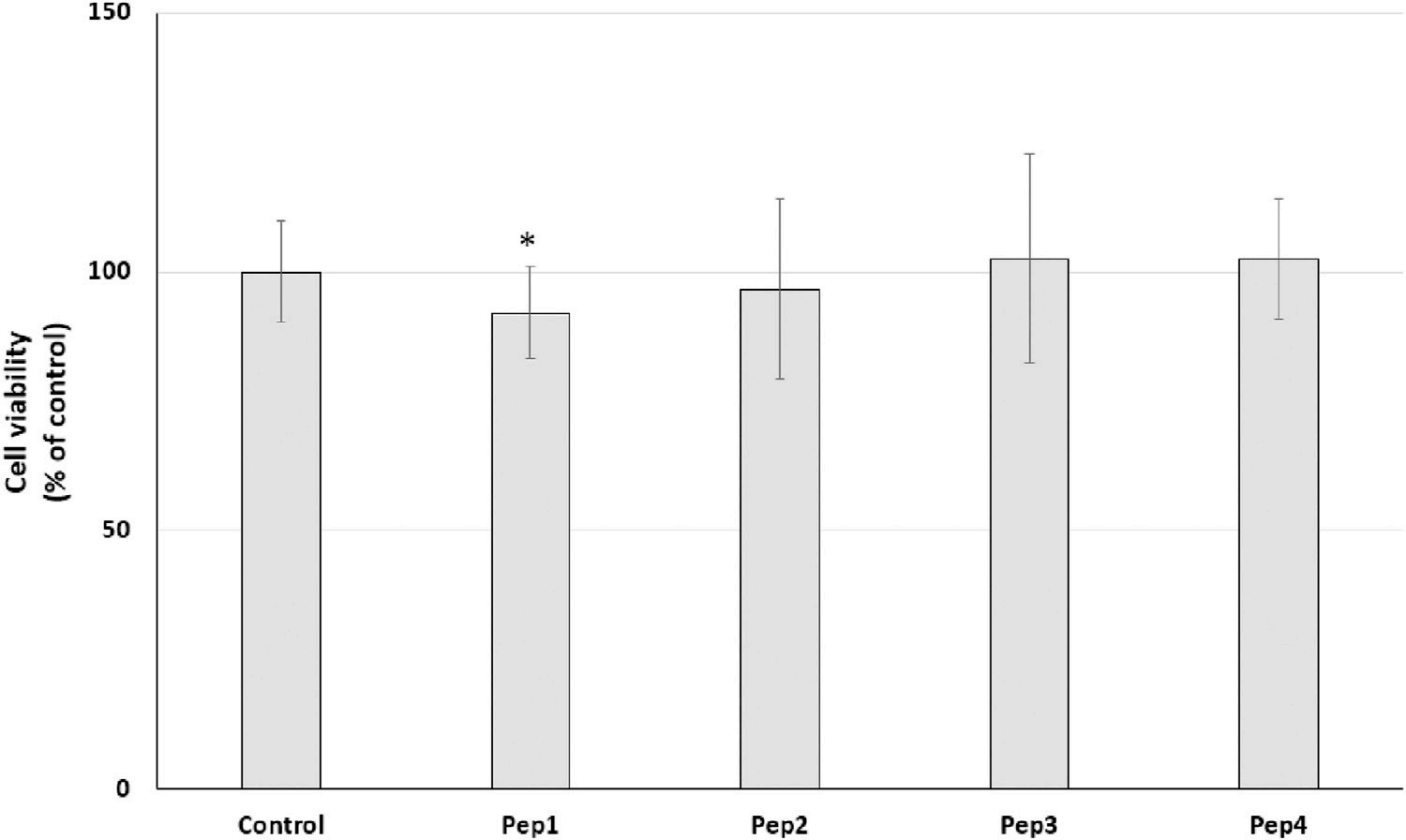
Effect of peptides (200 µM) on cell viability (%) expressed as resazurin reduction in the PANC-1 cell line after a 24-h exposure. Bar graph representing the mean and standard deviation values of two independent experiments, each consisting of six replicates, *p < 0.05 vs. control.

Our initial prediction using several databases revealed that at least Pep1 may have multifunctional activity and that Pep3 and Pep4 may have antimicrobial activity. Recently, many studies have indicated that bioactive peptides can have multifunctional activity (Diener et al., 2016; Jafari et al., 2022). Moreover, according to the AMP database (Wang et al., 2016), about 8% of the peptides are listed as anti-cancer peptides. Therefore, we decided to conduct a preliminary study to test the potential anti-cancer effect of the Pep1–Pep4 peptides. After 24 h exposure of MDA-MB-231 and PANC-1 cells to Pep1–Pep4 at a concentration of 200 μM, a statistically significant decrease in resazurin reduction compared to the control was observed only in PANC-1 cells after the addition of Pep1 to the medium (Figures 5, 6).

**FIGURE 7:**
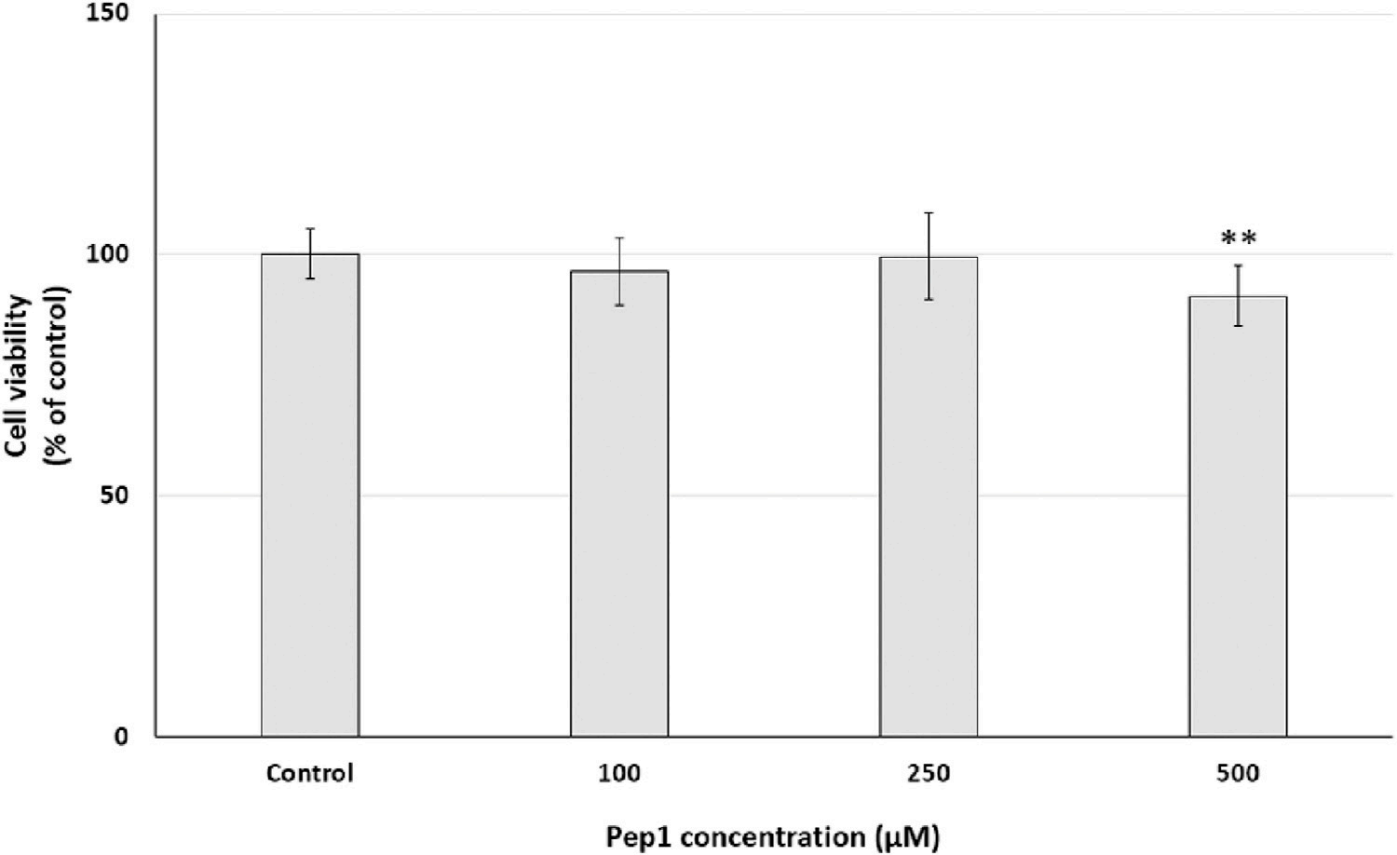
Effect of different Pep1 concentrations (100–500 µM) on cell viability (%) expressed as resazurin reduction in the PANC-1 cell line after a 24-h exposure. Bar graph representing the mean and standard deviation values of two independent experiments, each consisting of six replicates, **p < 0.01 vs. control.

To investigate the cell growth inhibitory effect of Pep1 on cancer cell lines in a dose-dependent manner, PANC-1 cells were treated with Pep1 at different concentrations of 100 µM–500 µM. As shown in Figure 7, after 24 h exposure of PANC-1 cells to the investigated peptide, a statistically significant decrease in resazurin reduction by 8.6% was observed compared to the control at 500 μM; no resazurin reduction was observed at concentrations of 100 and 250 µM. This suggests that Pep1 had a weak growth inhibitory effect on PANC-1 cells.

In view of the Boman index and the results of our studies, Pep1 could be a good template for further modifications to improve its activity against cancer cells (e.g., by incorporating unnatural amino acids into its sequence).

## 4 Conclusion

Milk is a rich source of bioactive compounds, including antimicrobial peptides. We have characterized zinc(II) and copper(II) complexes of four milk peptides that were the result of kombucha cleavage. Of the four peptides analyzed, two are composed of the same amino acids but in a different order: KFKGFVEPFPAVE (Pep3) and FVAPEPFVFGKEK (Pep4). The ITC results show that these two peptides form thermodynamically more stable complexes with Cu(II) at pH 6.1 than Pep1 and Pep2, which could most likely be due to the presence of the large aromatic phenylalanine ring in close proximity to the N-terminal metal-binding site. The cleaved peptides are not only the source of exciting bioinorganic chemistry but may also exhibit antioxidant and antimicrobial activities (especially Pep3 and Pep4).

We will test their activity against various bacteria and fungi, as well as their complexes, with copper in the near future. Prediction by different online tools has shown that all peptides are non-toxic and three of them are non-allergenic. In addition, they are not cytotoxic to normal human skin fibroblasts so they could be a good candidate for modification and further testing as a potential drug against skin diseases. In addition, using Pep1 is an attractive potential approach for cancer treatment. Our next goal is to develop peptoid analogs of these peptides to improve their activity, selectivity, and stability in serum. Further tests on other types of cancer cells are also desirable.

## Data Availability

The original contributions presented in the study are included in the article/Supplementary Material; further inquiries can be directed to the corresponding author.
