# Supplementary material for: Characterization of four peptides from milk fermented with kombucha cultures and their metal complexes—in search of new biotherapeutics
Source: Front Mol Biosci. 2024 Apr 4;11:1366588. doi: 10.3389/fmolb.2024.1366588 (PMC11024286; doi:10.3389/fmolb.2024.1366588)
Supplement: Supplementary file 1 [file DataSheet1.docx]

**Characterisation of four peptides from milk fermented with kombucha cultures, and their metal complexes - In search of new biotherapeutics.**

J. Kamińska,^1^ A. Hecel,^2^ J. Słowik,^1^ A. Rombel-Bryzek,^3^ M.Rowinska-Zyrek,^2^

D.Witkowska ^1*^

*^1^ Institute of Health Sciences, University of Opole, Poland,*

*Corresponding author e-mail:* [*danuta.witkowska@uni.opole.pl*](mailto:danuta.witkowska@uni.opole.pl)

*^2^ Faculty of Chemistry, University of Wroclaw, Wroclaw, Poland*

*^3^ Institute of Medical Sciences, University of Opole, Poland*


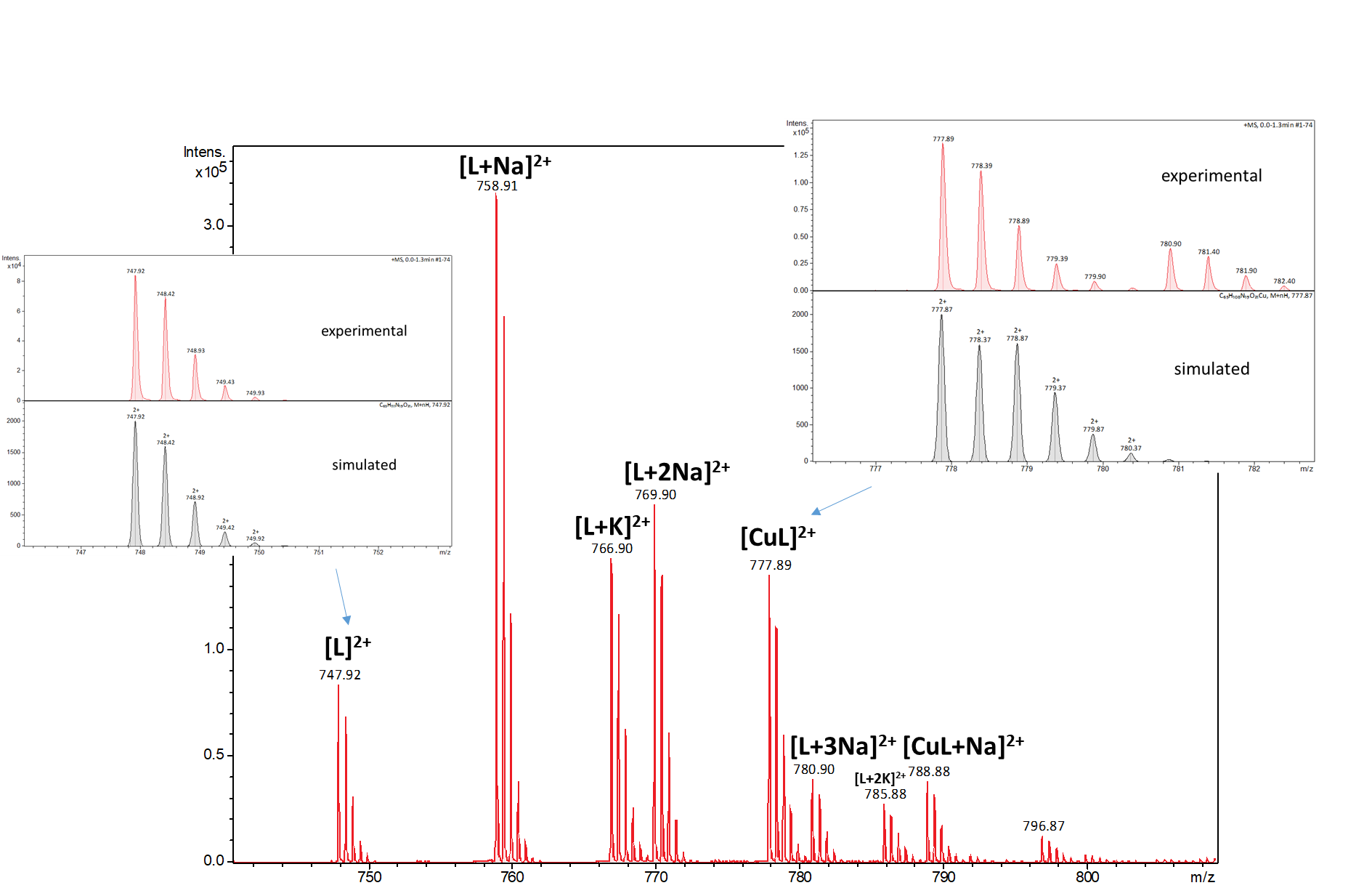

**Figure S1**. The upper panel show MS spectra of Cu(II)- AVPQEVLNENLLR (experimental and simulated) and the lower panel displays UV-Vis (left) and CD-spectroscopy (right) results of the Cu(II)-Pep1 titration over the pH range 3-11.


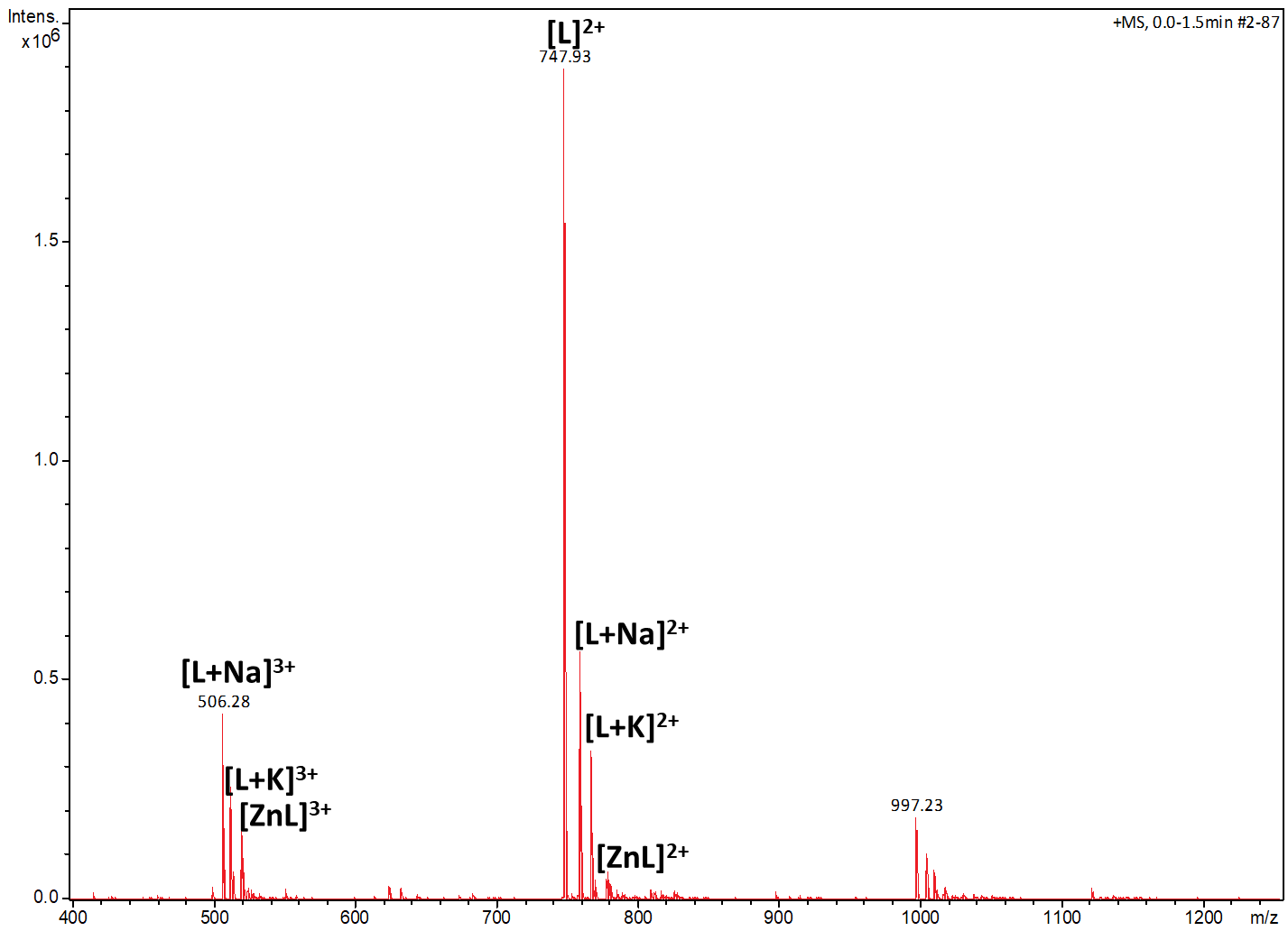


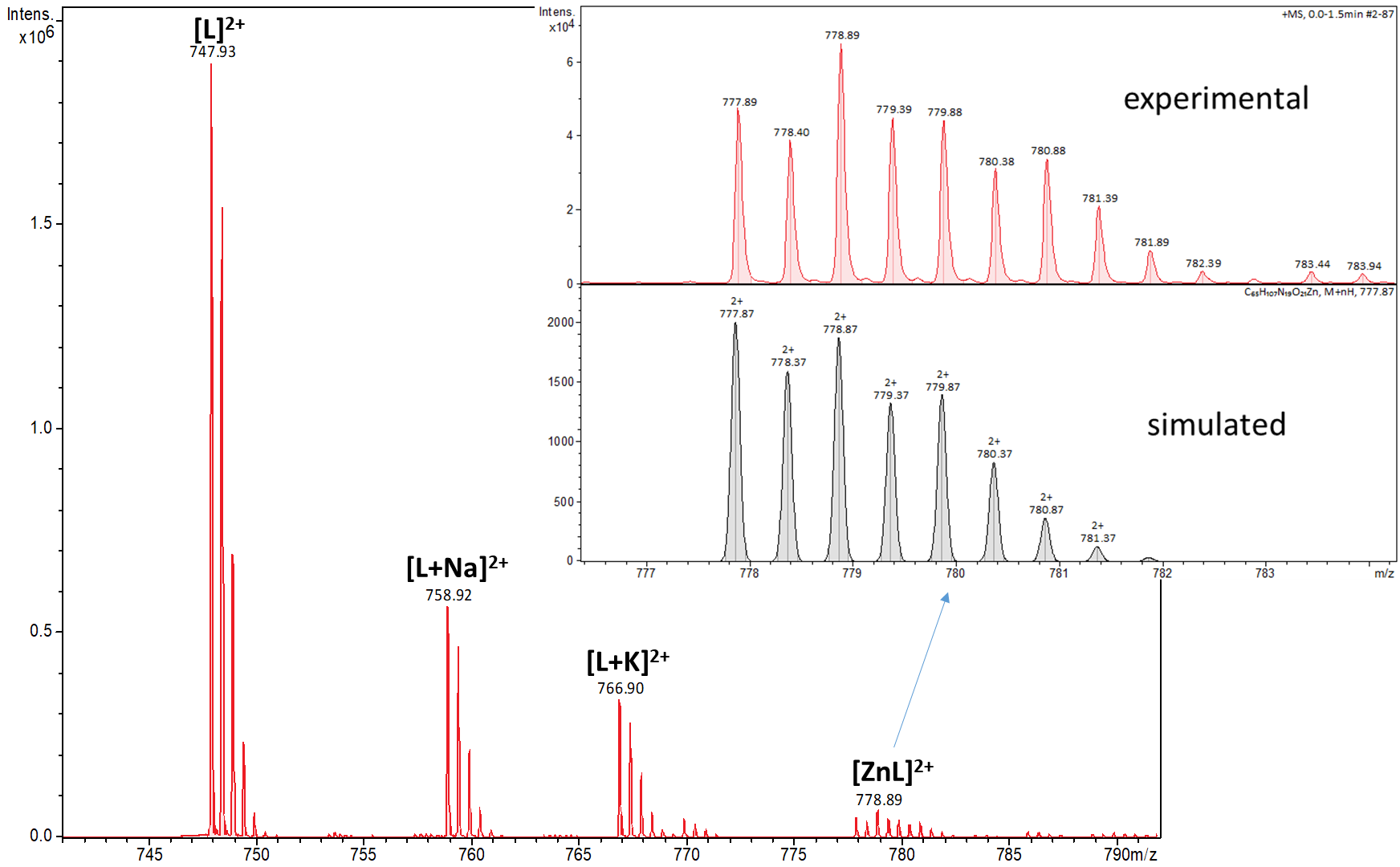


**Figure S2**. MS spectra of Zn (II)- AVPQEVLNENLLR. The upper panel shows the whole range and the lower panel displays chosen MS region (experimental and simulated)

A


 B

**Figure S3.** Distribution diagrams for the formation of: A. Cu(II) complexes with Pep1; B. Zn(II) complexes with Pep1.


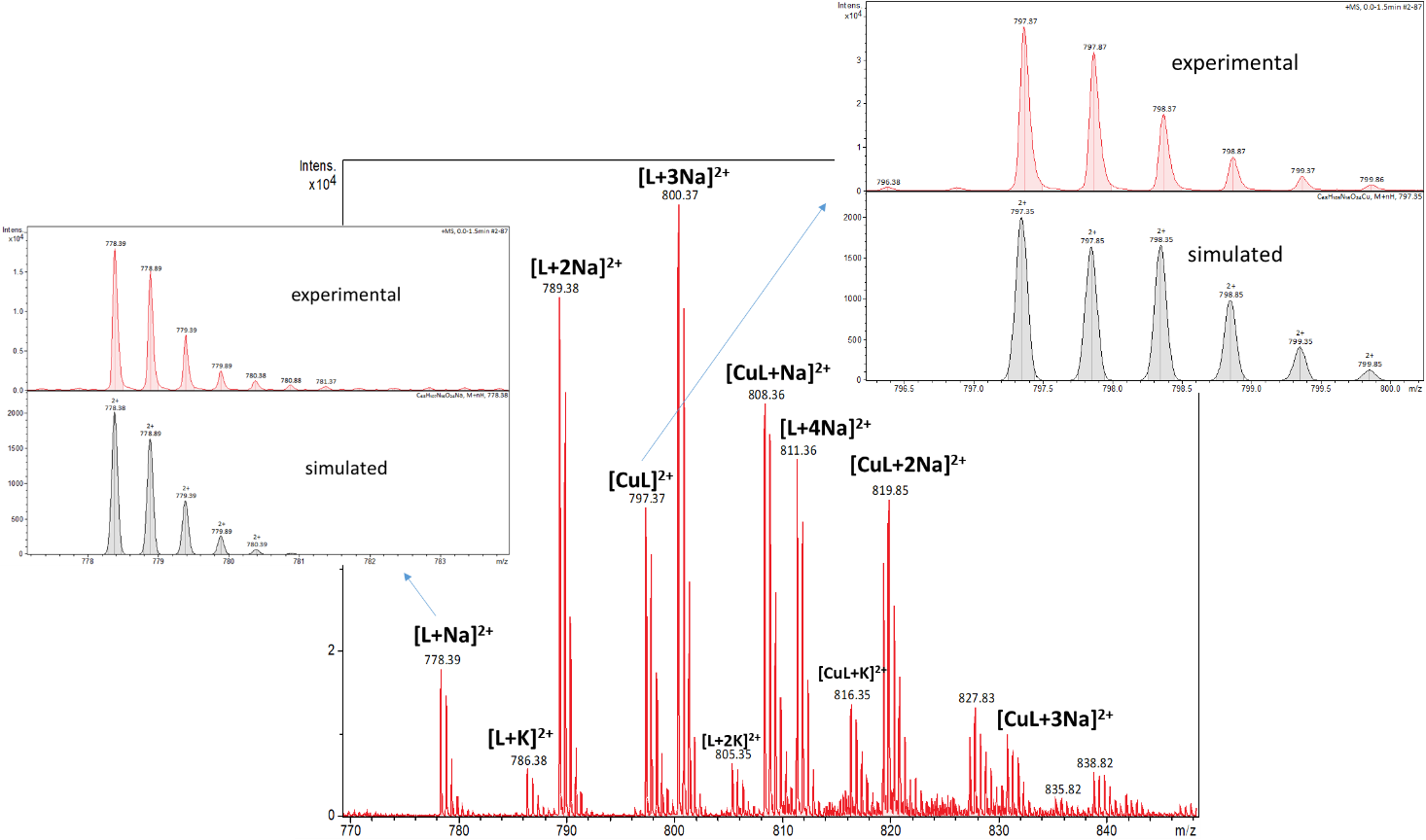

**Figure S4**. The upper panel show MS spectra of Cu(II)-**YLQGSNLVVPLTDD** (experimental and simulated) and the lower panel displays UV-Vis (left) and CD-spectroscopy (right) results of the Cu(II)-Pep2 titration over the pH range 3-11.


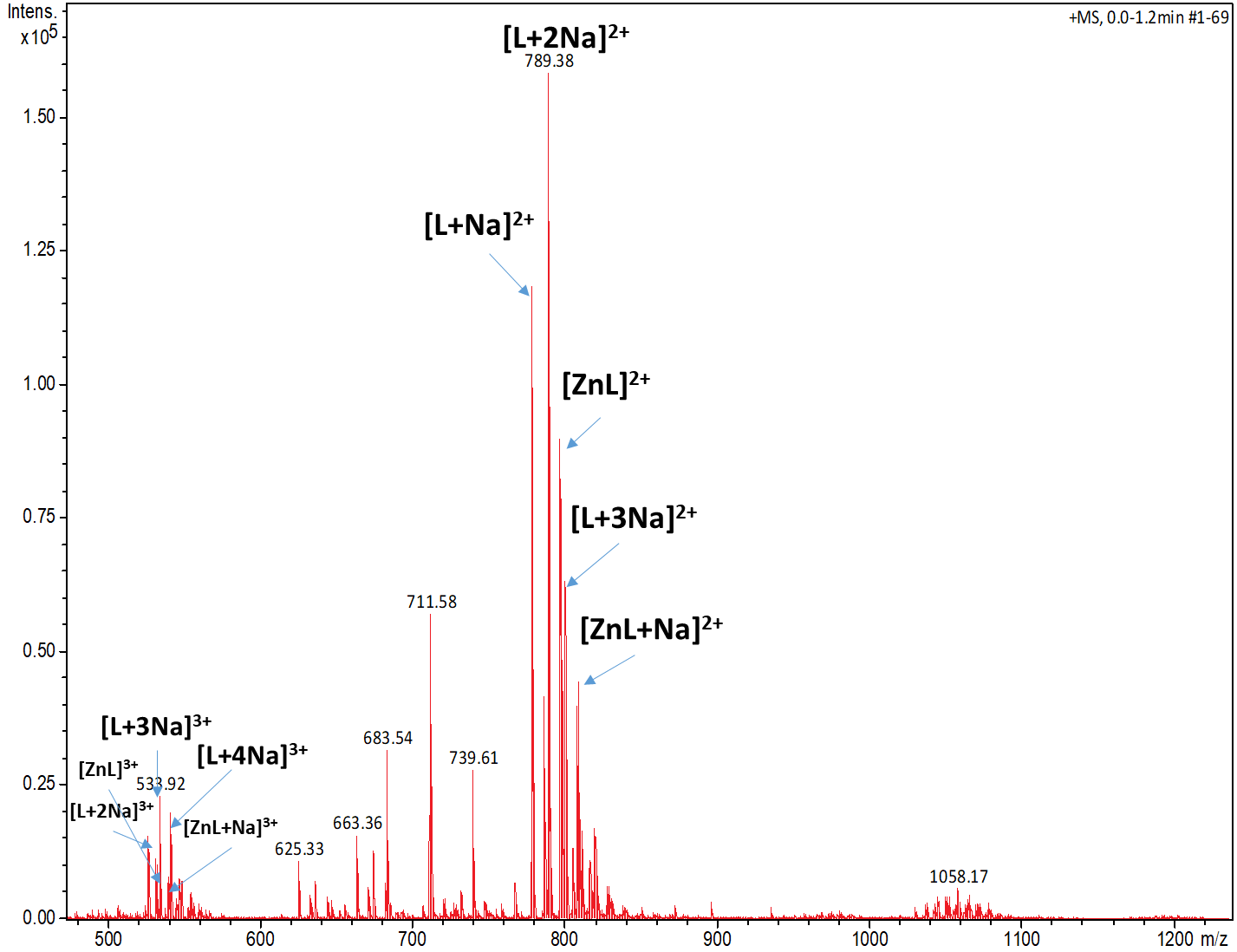


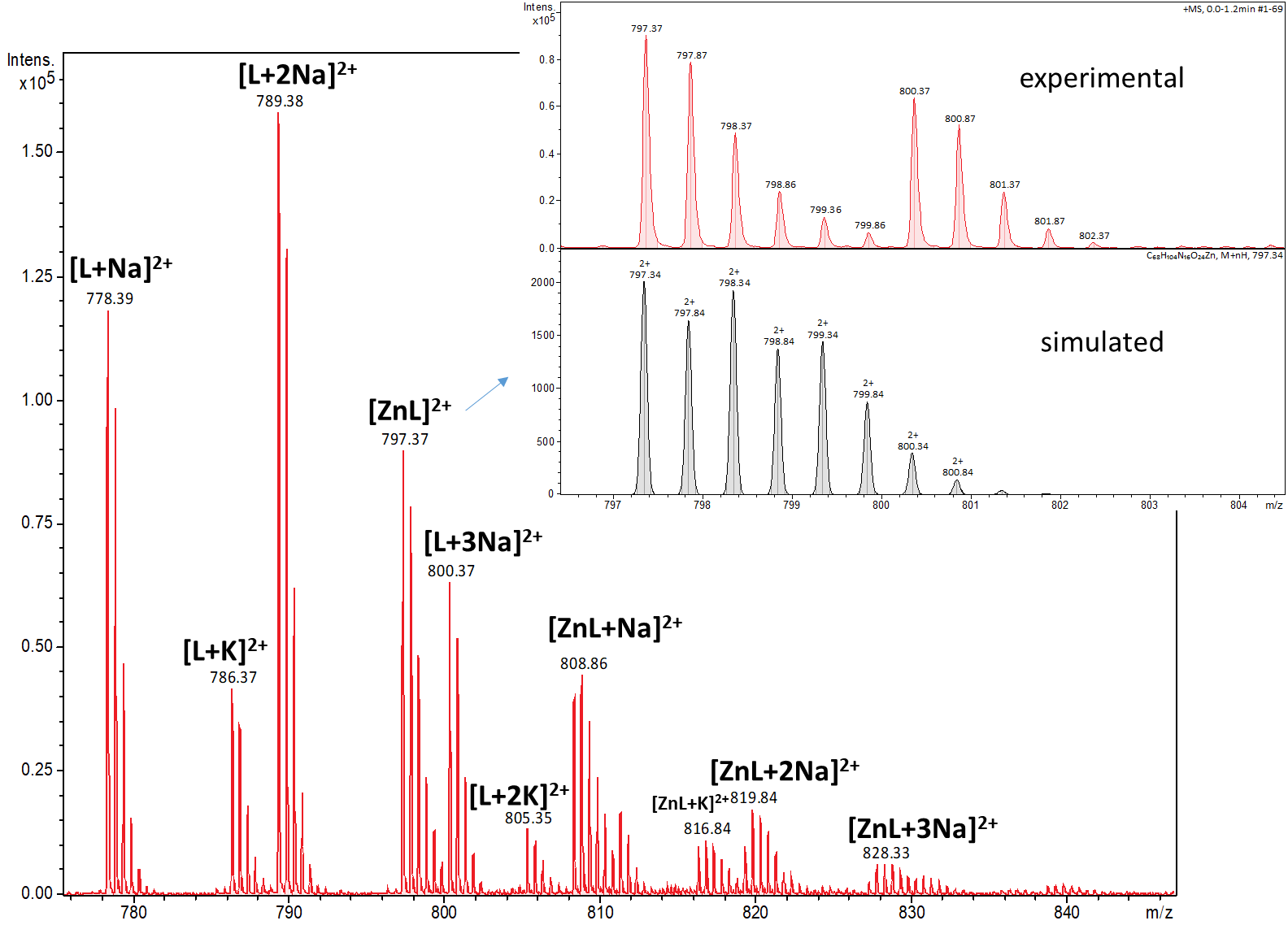


**Figure S5**. MS spectra of Zn (II)- **YLQGSNLVVPLTDD.** The upper panel shows the whole range and the lower panel displays chosen MS region (experimental and simulated)

A

 B

**Figure S6.** Distribution diagrams for the formation of: A. Cu(II) complexes with Pep2; B. Zn(II) complexes with Pep2.


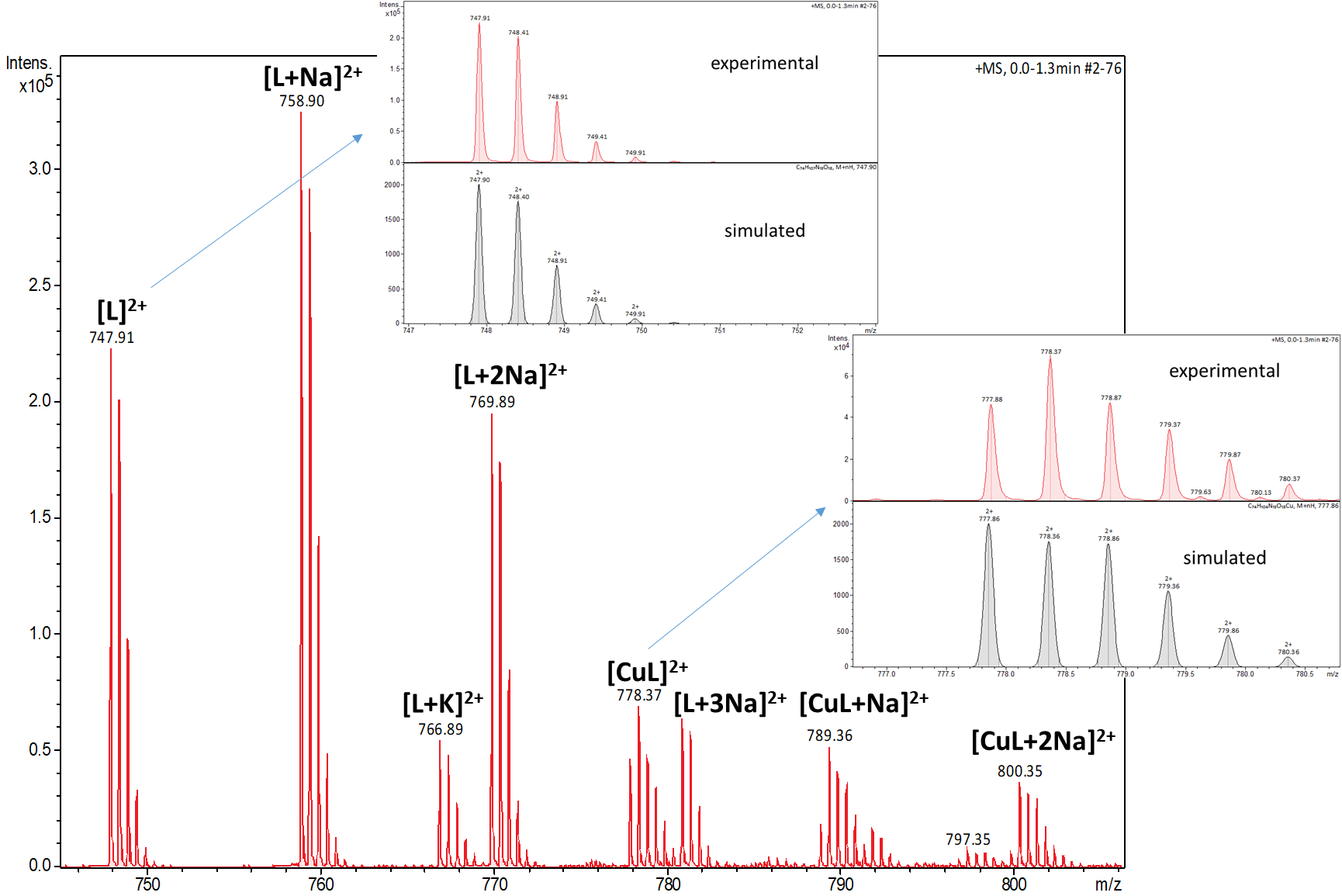

**Figure S7**. The upper panel show MS spectra of Cu(II)- **KFKGFVEPFPAVE** (experimental and simulated) and the lower panel displays UV-Vis (left) and CD-spectroscopy (right) results of the Cu(II)-Pep3 titration over the pH range 3-11.


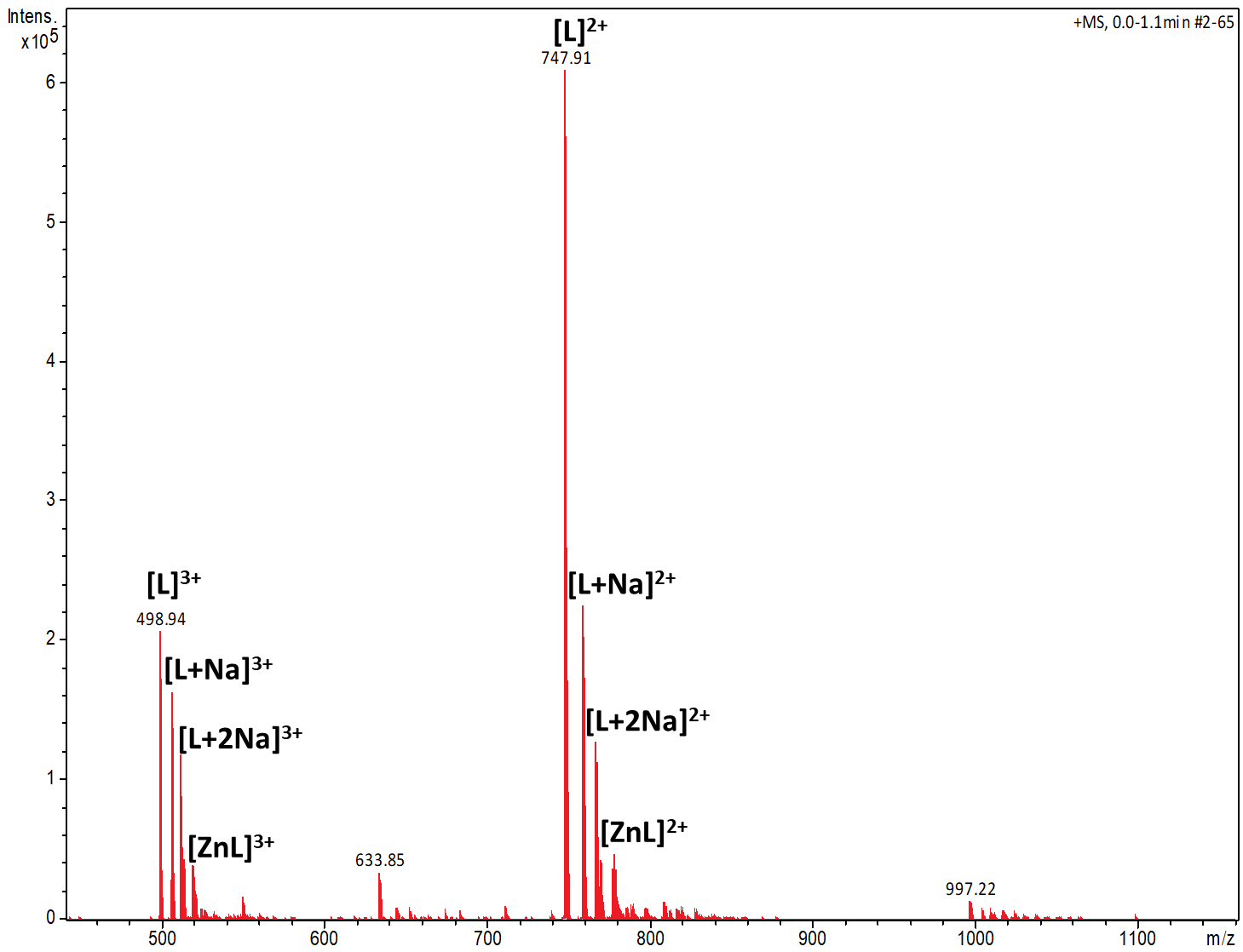


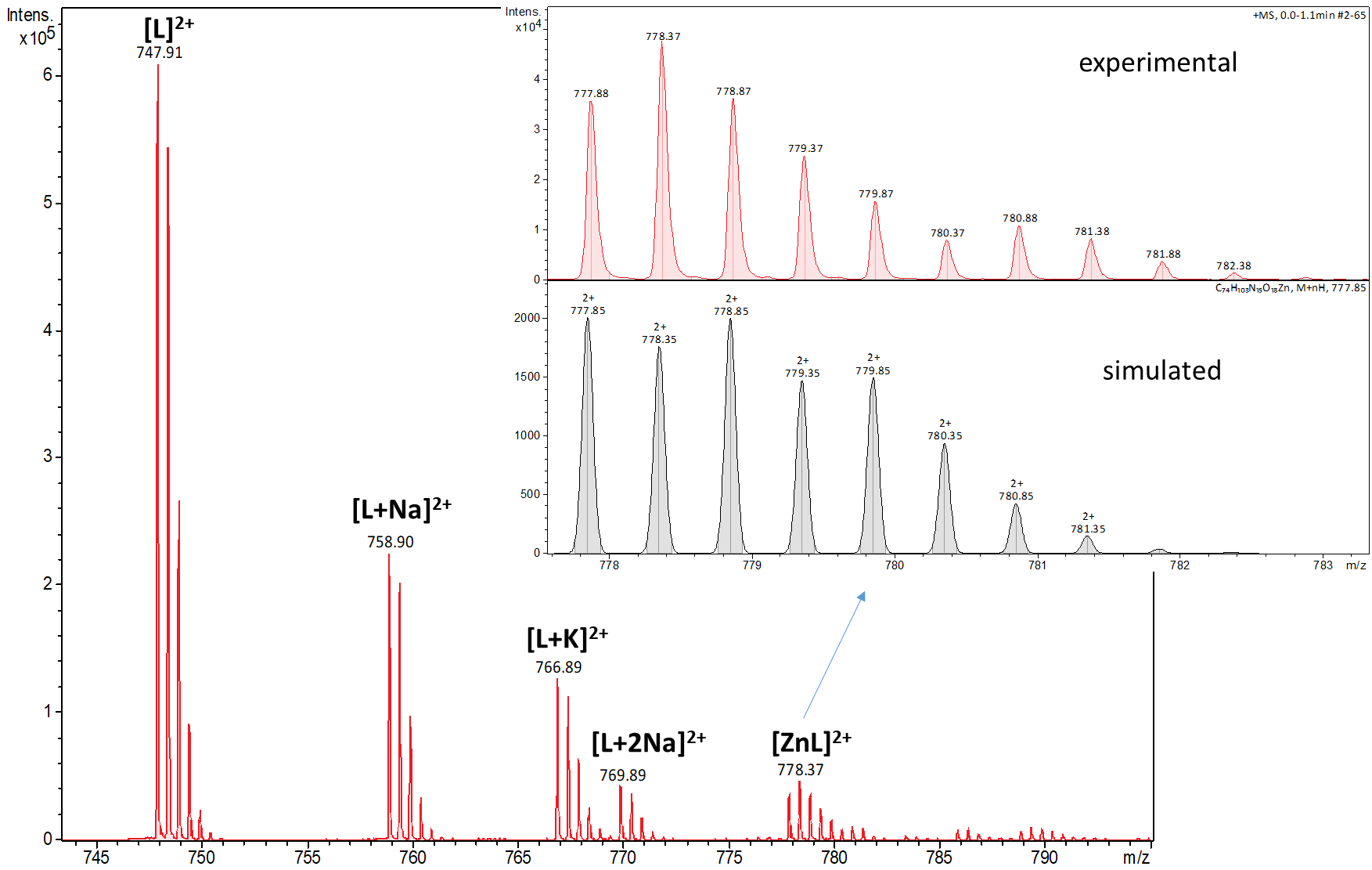


**Figure S8**. MS spectra of Zn (II)- **KFKGFVEPFPAVE.** The upper panel shows the whole range and the lower panel displays chosen MS region (experimental and simulated)

 A

 B

**Figure S9.** Distribution diagrams for the formation of: A. Cu(II) complexes with Pep3; B. Zn(II) complexes with Pep3.


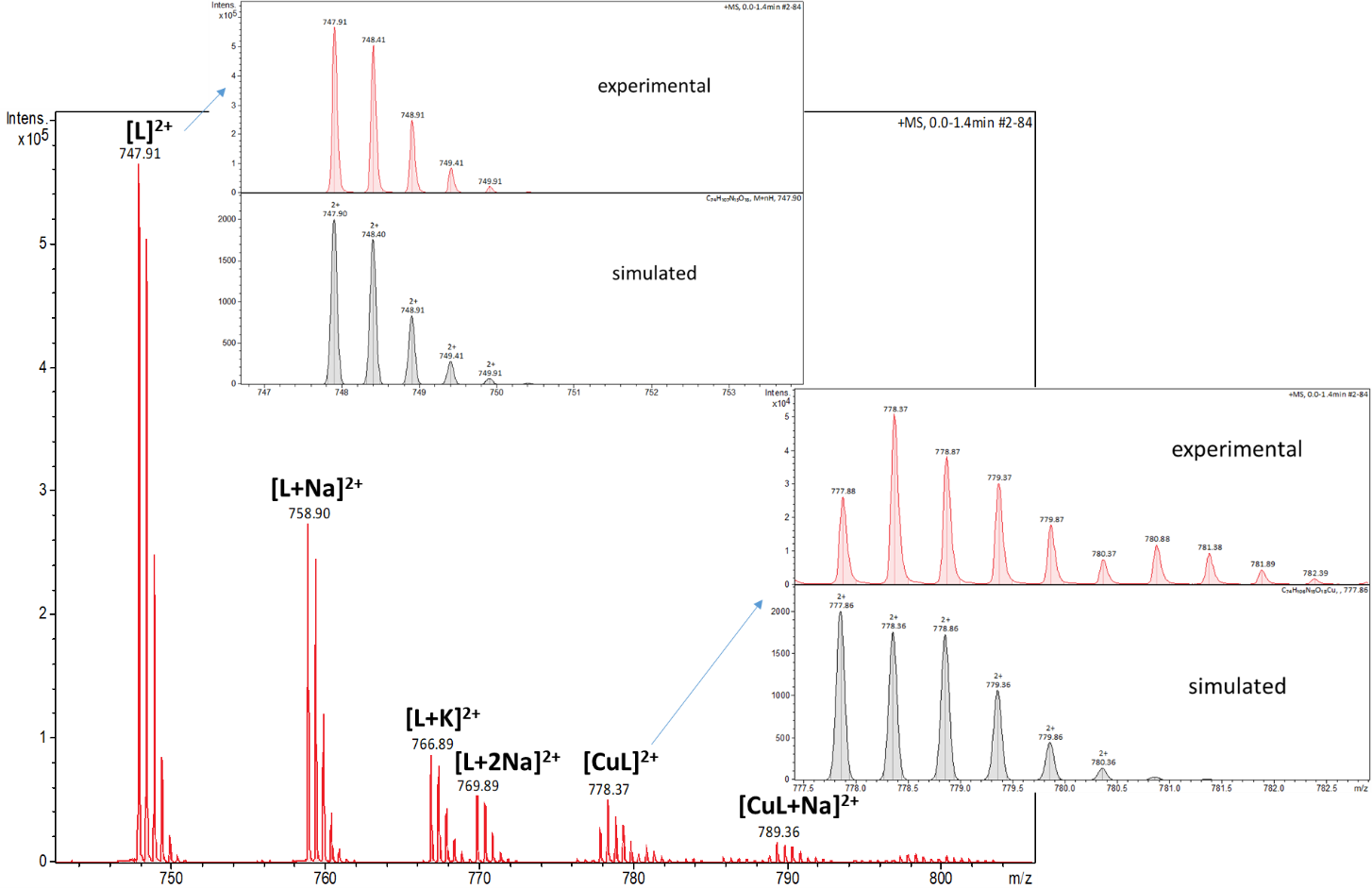

**Figure S10**. The upper panel show MS spectra of Cu(II)- **FVAPEPFVFGKEK**(experimental and simulated) and the lower panel displays UV-Vis (left) and CD-spectroscopy (right) results of the Cu(II)-Pep4 titration over the pH range 3-11.


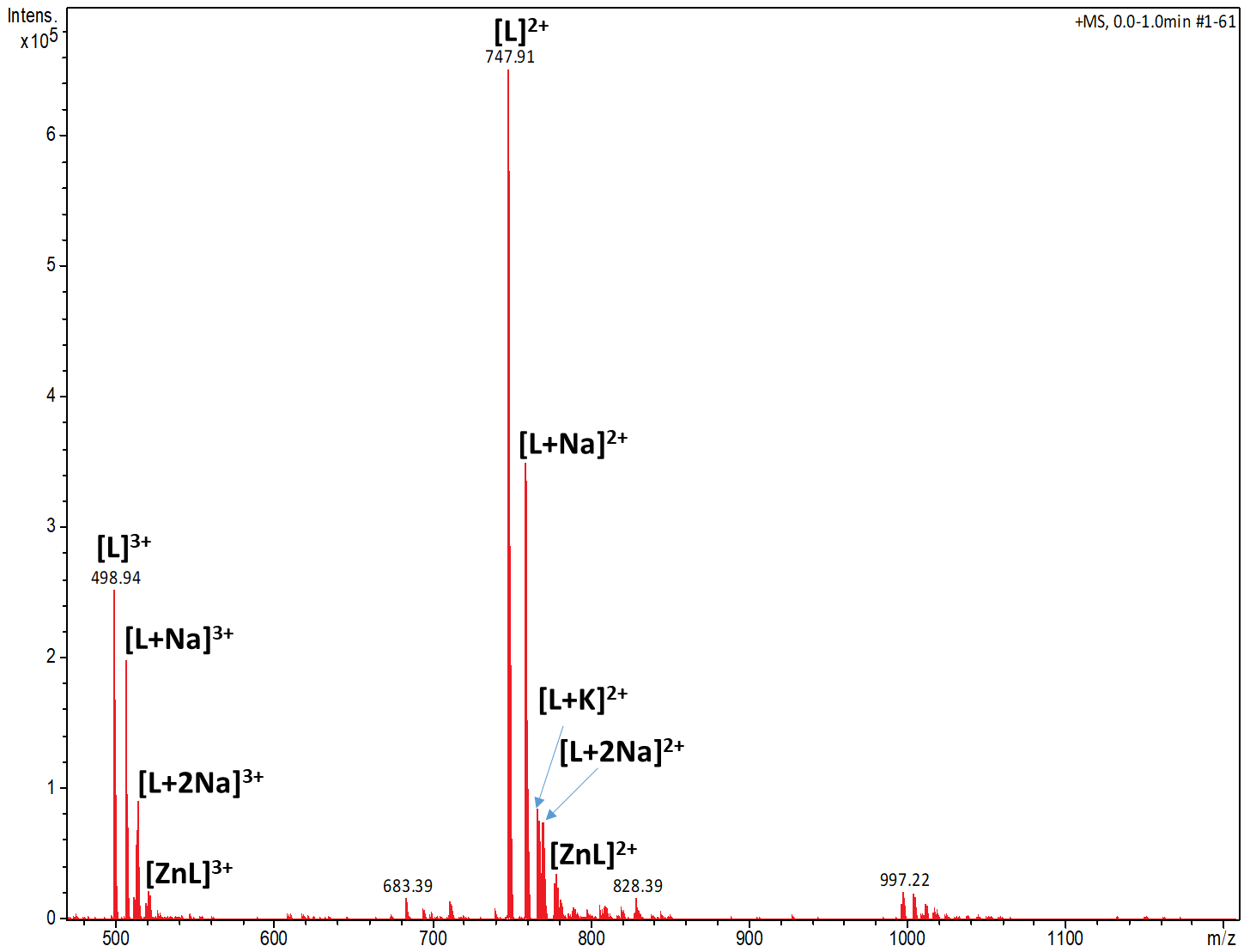


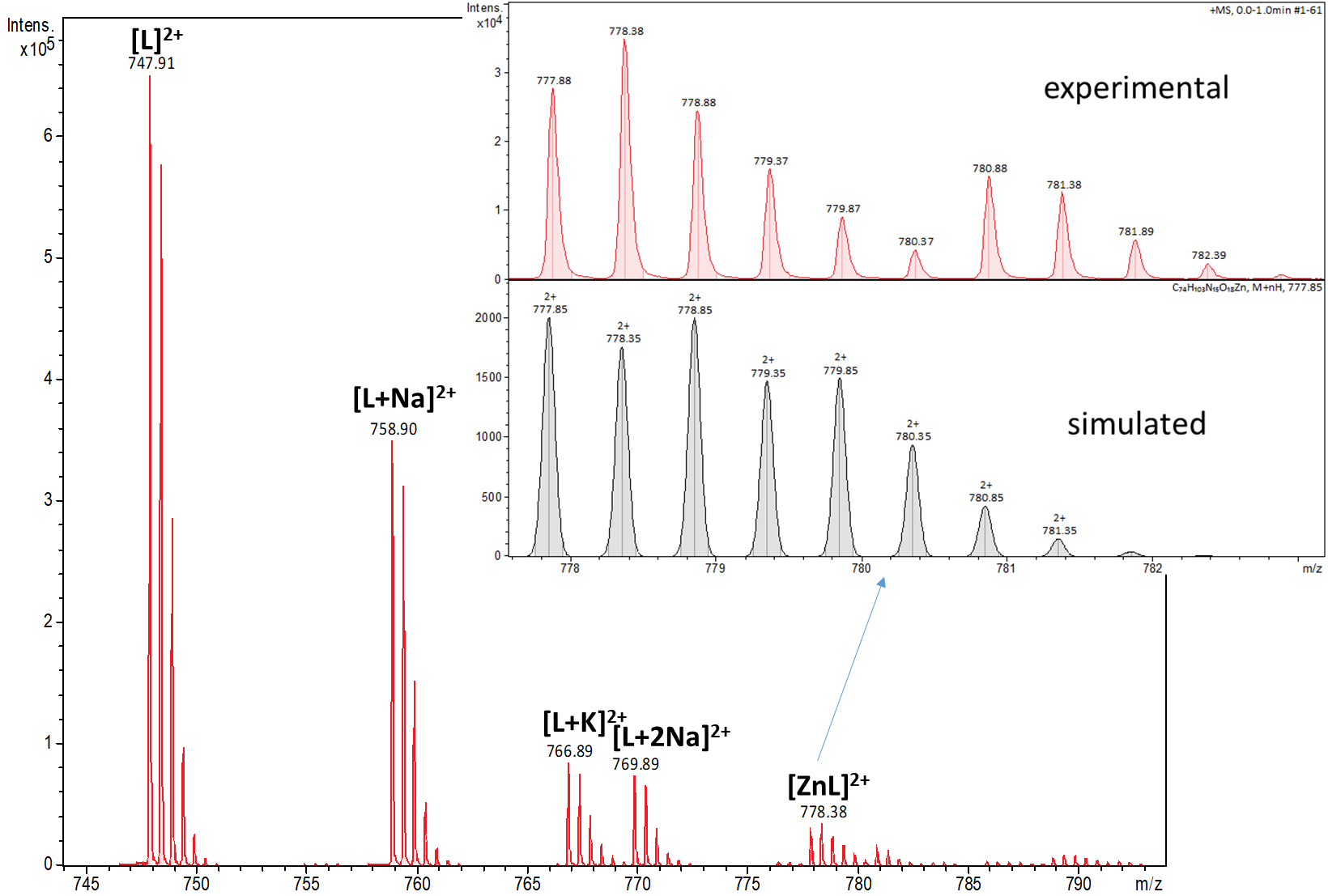


**Figure S11**. MS spectra of Zn (II)- **FVAPEPFVFGKEK**. The upper panel shows the whole range and the lower panel displays chosen MS region (experimental and simulated)

 A

 B

**Figure S12.** Distribution diagrams for the formation of: A. Cu(II) complexes with Pep4; B. Zn(II) complexes with Pep4.

# Figure S13. ITC traces and binding isotherms of titration of the Pep1-Pep4 solutions with Zn(II) ions (c = 6mM).

**Table S1**. Potentiometric data for proton and Zn(II) complexes with Pep1. Titrations were carried out over the pH range 2–11 at T = 298 K in an aqueous solution with 4 mM HClO_4_ and 0.1 M NaClO_4_ . Standard deviations are shown in brackets.

| **species** | **logβ** | **pKa** | **Proposed donors** |
| --- | --- | --- | --- |
| HL | 7.94(2) | 7.94 (N-terminal NH_3_^+^) |  |
| H_2_L | 12.78(3) | 4.84 (E) |  |
| H_3_L | 16.66(3) | 3.88 (E) |  |
| H_4_L | 19.17(5) | 2.51 (C-terminal COOH) |  |
| ZnH_2_L | 18.67(6) |  |  |
| ZnHL | 14.57(6) | 4.10 | E |
| ZnL | 9.87(8) | 4.70 | NH_2_ |
|  |  |  | NH_2_,H_2_O |
| ZnH_-2_L | -5.90(9) |  | NH_2_,2H_2_O |
| ZnH_-3_L | -13.78(9) | 7.88 | NH_2_,3H_2_O |

**Table S2**. Potentiometric data for proton and Zn(II) complexes with Pep2. Titrations were carried out over the pH range 2–11 at T = 298 K in an aqueous solution with 4 mM HClO_4_ and 0.1 M NaClO_4_ . Standard deviations are shown in brackets.

| **species** | **logβ** | **pKa** | **proposed donors** |
| --- | --- | --- | --- |
| HL | 9.71(1) | 9.71(Y) |  |
| H_2_L | 16.92(2) | 7.21(N-terminal NH_3_^+^) |  |
| H_3_L | 21.87(3) | 4.95(D) |  |
| H_4_L | 25.79(3) | 3.92(D) |  |
| H_5_L | 28.33(5) | 2.54(C-terminal COOH) |  |
| ZnH_3_L | 26.91(2) |  |  |
| ZnH_2_L | 22.73(4) | 4.18 | D |
| ZnHL | 17.66(6) | 5.07 | NH_2_ |
| ZnL | 9.86(8) | 7.80 | NH_2_,H_2_O |
| ZnH_-1_L | - |  | NH_2_,2H_2_O |
| ZnH_-2_L | -5.92(6) |  | NH_2_,3H_2_O |
| ZnH_-3_L | -15.54(7) | 9.62 | NH_2_,3H_2_O |

**Table S3.** Potentiometric data for proton and Zn(II) complexes with Pep3. Titrations were carried out over the pH range 2–11 at T = 298 K in an aqueous solution with 4 mM HClO_4_ and 0.1 M NaClO_4_ . Standard deviations are shown in brackets.

| **species** | **logβ** | **pKa** | **Proposed donors** |
| --- | --- | --- | --- |
|  |  |  |  |
| HL | 11.00(1) | 11.00(K) |  |
| H_2_L | 20.94(1) | 9.94(K) |  |
| H_3_L | 28.11(2) | 7.17(N-terminal NH_3_^+^) |  |
| H_4_L | 32.92(2) | 4.81 (E) |  |
| H_5_L | 36.93(2) | 4.01 (E) |  |
| H_6_L | 39.76(2) | 2.83 (C-terminal COOH) |  |
| ZnH_3_L | 33.45(7) |  |  |
| ZnH_2_L | 28.36(9) | 5.09 | NH_2_ |
| ZnHL | 21.07(9) | 7.29 | NH_2_,H_2_O |
| ZnL | - |  | NH_2_,2H_2_O |
| ZnH_-1_L | - |  | NH_2_,3H_2_O |
| ZnH_-2_L | -2.69(9) |  | NH_2_,4H_2_O |

**Table S4.** Potentiometric data for proton and Zn(II) complexes with Pep4. Titrations were carried out over the pH range 2–11 at T = 298 K in an aqueous solution with 4 mM HClO_4_ and 0.1 M NaClO_4_ . Standard deviations are shown in brackets.

| **species** | **logβ** | **pKa** | **Proposed donors** |
| --- | --- | --- | --- |
| HL | 10.61(1) | 10.61 (K) |  |
| H_2_L | 20.22(1) | 9.61(K) |  |
| H_3_L | 27.49(3) | 7.27 (N-terminal NH_3_^+^) |  |
| H_4_L | 32.35(4) | 4.86 (E) |  |
| H_5_L | 36.33(4) | 3.98 (E) |  |
| ZnH_3_L | 33.93(6) |  |  |
| ZnH_2_L | 29.66(5) | 4.27 | NH_2_ |
| ZnHL | 24.63(8) | 5.03 | NH_2_,H_2_O |
| ZnL | 16.72(9) | 7.91 | NH_2_,2H_2_O |
| ZnH_-1_L | - |  | NH_2_,3H_2_O |
| ZnH_-2_L | 1.49(8) |  | NH_2_,4H_2_O |
